# Proteome Profiling Outperforms Transcriptome Profiling for Coexpression Based Gene Function Prediction*[Fn FN2]

**DOI:** 10.1074/mcp.M116.060301

**Published:** 2016-11-11

**Authors:** Jing Wang, Zihao Ma, Steven A. Carr, Philipp Mertins, Hui Zhang, Zhen Zhang, Daniel W. Chan, Matthew J. C. Ellis, R. Reid Townsend, Richard D. Smith, Jason E. McDermott, Xian Chen, Amanda G. Paulovich, Emily S. Boja, Mehdi Mesri, Christopher R. Kinsinger, Henry Rodriguez, Karin D. Rodland, Daniel C. Liebler, Bing Zhang

**Affiliations:** From the ‡Department of Biomedical Informatics, Vanderbilt University Medical Center, Nashville, Tennessee 37232;; §Lester and Sue Smith Breast Center, Baylor College of Medicine, Houston, Texas 77030;; ¶Department of Molecular and Human Genetics, Baylor College of Medicine, Houston, Texas 77030;; ‖Broad Institute of MIT and Harvard, Cambridge, Massachusetts 02142;; **Department of Pathology, Johns Hopkins Medical Institutions, Baltimore, Maryland 21205;; ‡‡Department of Medicine, Baylor College of Medicine, Houston, Texas 77030;; §§Department of Internal Medicine, Washington University School of Medicine, St. Louis, Missouri 63110;; ¶¶Biological Sciences Division, Pacific Northwest National Laboratory, Richland, Washington 99352;; ‖‖University of North Carolina at Chapel Hill, 130 Mason Farm Road, Chapel Hill, North Carolina 27599;; *^a^*Clinical Research Division, Fred Hutchinson Cancer Research Center, 1100 Eastlake Avenue East, Seattle, Washington 98109;; *^b^*Office of Cancer Clinical Proteomics Research, National Cancer Institute, Bethesda, Maryland 20892;; *^c^*Department of Biochemistry, Vanderbilt University, Nashville, Tennessee 37232;; *^d^*Jim Ayers Institute for Precancer Detection and Diagnosis, Vanderbilt-Ingram Cancer Center, Nashville, Tennessee 37232

## Abstract

Coexpression of mRNAs under multiple conditions is commonly used to infer cofunctionality of their gene products despite well-known limitations of this “guilt-by-association” (GBA) approach. Recent advancements in mass spectrometry-based proteomic technologies have enabled global expression profiling at the protein level; however, whether proteome profiling data can outperform transcriptome profiling data for coexpression based gene function prediction has not been systematically investigated. Here, we address this question by constructing and analyzing mRNA and protein coexpression networks for three cancer types with matched mRNA and protein profiling data from The Cancer Genome Atlas (TCGA) and the Clinical Proteomic Tumor Analysis Consortium (CPTAC). Our analyses revealed a marked difference in wiring between the mRNA and protein coexpression networks. Whereas protein coexpression was driven primarily by functional similarity between coexpressed genes, mRNA coexpression was driven by both cofunction and chromosomal colocalization of the genes. Functionally coherent mRNA modules were more likely to have their edges preserved in corresponding protein networks than functionally incoherent mRNA modules. Proteomic data strengthened the link between gene expression and function for at least 75% of Gene Ontology (GO) biological processes and 90% of KEGG pathways. A web application Gene2Net (http://cptac.gene2net.org) developed based on the three protein coexpression networks revealed novel gene-function relationships, such as linking ERBB2 (HER2) to lipid biosynthetic process in breast cancer, identifying PLG as a new gene involved in complement activation, and identifying AEBP1 as a new epithelial-mesenchymal transition (EMT) marker. Our results demonstrate that proteome profiling outperforms transcriptome profiling for coexpression based gene function prediction. Proteomics should be integrated if not preferred in gene function and human disease studies.

Cellular functions require coordinated expression of genes involved in the same biological pathways or protein complexes. High-throughput mRNA profiling has been the dominant approach to studying gene expression and its relationship to cellular functions. Coexpression of mRNAs under multiple conditions is commonly used to infer cofunctionality of their gene products (1), and this “guilt-by-association” (GBA)^1^ heuristic is the basis for analyzing mRNA profiling data using gene clustering (2), coexpression network analysis (3–5), and pathway and gene set enrichment analysis (6–8). However, genes with similar mRNA expression profiles are not necessarily functionally coupled due to reasons such as transcriptional leakage and nonspecific occurrence of *cis*-regulatory elements in the genome (9–11). Distinguishing accidental transcriptional covariation from those that are functionally important is a well-known challenge, and strategies such as meta-analysis (12) and evolutionary constraint (10, 13) have been developed to address this challenge.

Recent advancements in mass spectrometry-based proteomic technologies have enabled global expression profiling at the protein level, and the concordance between mRNA and protein profiling data has been extensively studied during the past decade (14, 15). Although a few publications suggest that gene expression is mostly controlled at the mRNA level (16–18), many studies have reported a considerable discrepancy between mRNA and protein profiles in human and other model organisms (15, 19–22). It is not completely clear how much of the reported mRNA-protein discrepancy is due to technological issues and how much is due to underlying biology. Importantly, whether proteome profiling data can outperform transcriptome profiling data for coexpression based gene function prediction is largely unknown.

The deep proteome profiling data sets recently generated by the Clinical Proteomic Tumor Analysis Consortium (CPTAC) on the breast (23), colorectal (22), and ovarian (24) tumors that had been transcriptomically profiled by The Cancer Genome Atlas (TCGA) (25–27) provided a new opportunity to address this question. We constructed gene coexpression networks based on mRNA and protein profiling data sets, respectively, for each of the three cancer types. Comprehensive comparisons between the mRNA and protein coexpression networks constructed for the same cancer type allowed us to systematically investigate the relative utility of mRNA and protein profiling data in predicting gene cofunctionality.

## MATERIALS AND METHODS

### 

#### 

##### Protein and mRNA Profiling Data

##### Breast Cancer

The gene-level proteomics data for breast cancer was downloaded from Mertins *et al.* (23). An isobaric peptide labeling approach (iTRAQ) was employed to quantify protein levels. Protein quantification was based on iTRAQ reporter ion ratios to the internal standard. Data normalization was performed using a 2-component Gaussian mixture model-based normalization algorithm. The data set contained 9988 genes and 77 samples. Only the 6281 genes without any missing values across all samples were included in this study. The gene-level RNA-Seq data was downloaded from the Firehose website (http://gdac.broadinstitute.org), which was from the Illumina HiSeq 2000 RNA Sequencing Version 2 analysis and was normalized by the RSEM algorithm (28). The RNA-Seq data set included 20501 genes and 1058 samples. The two data sets had 5988 overlapping genes and 77 overlapping samples. Only overlapping samples and genes were included in this study, and this was also true for the other two cancer types.

##### Colorectal Cancer

The gene-level proteomics data for colorectal cancer was downloaded from Zhang *et al.* (22). Label-free shotgun proteomics was used to quantify protein levels. Protein quantification was based on spectral counts, which were quantile normalized followed by log-transformation. The data set contained 3899 genes and 90 samples. The gene level RNASeq data normalized by the RSEM algorithm was downloaded from the Firehose website (http://gdac.broadinstitute.org), which contained 20501 genes and 264 samples. There were 3764 overlapping genes and 87 overlapping samples between the two data sets.

##### Ovarian Cancer

The gene-level proteomics data for ovarian cancer was downloaded from Zhang *et al.* (24). Similar to the breast cancer data set, protein quantification was based on iTRAQ reporter ion ratios to the internal standard. Data normalization was performed using a global median centering algorithm. The data set contained 4186 genes across all 174 samples. Only the 3327 genes with low technical variance and without any missing values across all samples were included in this study. The gene-level microarray data was downloaded from the Firehose website (http://gdac.broadinstitute.org), which was from the Agilent 244K platform and was normalized by the lowess normalization method (29). The microarray data set contained 17814 genes and 541 samples. The two data sets had 2988 overlapping genes and 174 overlapping samples.

##### Identification of Functionally Similar and Dissimilar Gene Pairs

Gene Ontology (GO) based semantic similarity was computed for all gene pairs to identify functionally similar and dissimilar gene pairs.

##### GO

The GO vocabulary and annotation data were downloaded from the GO website (www.geneontology.org) in November 2014. To ensure high quality of the annotations, we excluded those denoted as IEA (Inferred from Electronic Annotation) or ND (No biological Data available) (30).

##### Calculation of Similarity Scores for Pairs of GO Terms

The Resnik similarity score (31) was computed to measure the similarity between each pair of GO terms. Specifically, the information content of a term *c* was defined as *IC*(*c*) = -log(*p*(*c*)), where *p*(*c*) is the number of genes annotated to the term and its descendants divided by the number of all genes annotated to the corresponding root term (*i.e.* biological process, cellular component, or molecular function). Let *P*(*m*,*n*) represents the set of common ancestor terms of terms *m* and *n*, the Resnik similarity score between *m* and *n* was calculated as:

$$Sim_{resnik}\left(m,n\right)=\max_{\text{c}\epsilon P\left(m,n\right)}\left[IC\left(c\right)\right]$$

##### Calculation of Similarity Scores for Pairs of Genes

The similarity scores for term pairs describing two genes were combined to calculate the semantic similarity score of the two genes based on the best-match average (BMA) approach (32). Let *A_1_* and *A_2_* be the sets of annotation terms for genes *G_1_* and *G_2_*, respectively, and *#G_1_* and *#G_2_* be the numbers of terms included in *A_1_* and *A_2_*, respectively. The BMA score for the two genes was defined as

$$BMA\left(G_{1},\;G_{2}\right)=\frac{S\left(G_{1},\;G_{2}\right)+S\left(G_{2},\;G_{1}\right)}{\#G_{1}+\#G_{2}}$$

where *S*(*G*_1_, *G*_2_) = Σ_*m*ϵ*A*_1__ *max*_*n*ϵ*A*_2__(*sim*(*m*, *n*)) and *S(G*_2,_ *G*_1_) = Σ_*m*ϵ*A*_2__ *max*_*n*ϵ*A*_1__(*sim*(*m*, *n*)).

All gene pairs were ranked from the highest BMA score to the lowest BMA score. The top 1%, 5%, 10%, 15%, 20% and 25% gene pairs in the ranked list were selected as candidate gold standard sets of functionally similar gene pairs whereas the bottom 1%, 5%, 10%, 15%, 20% and 25% were selected as candidate gold standard sets of functionally dissimilar gene pairs.

##### Comparison of Different Methods for Coexpression Network Construction Using mRNA Profiling Data Sets

To select a superior methodology for coexpression network construction, we compared three methods that are widely used for mRNA coexpression network construction, including: the value-based method (33), the *K*-nearest neighbor method (34), and the ARACNE (Algorithm for the Reconstruction of Accurate Cellular Networks) method (35). The mRNA profiling data sets for the three cancer types after overlapping with corresponding proteomics data sets as described above were used for method comparison. We only included positive correlations in our analysis because we found that a strong negative correlation between two genes did not necessarily correspond to high functional similarity between the genes (supplemental Fig. S1), which is consistent with previous reports (36, 37).

##### Method Description

In the value-based method (33), spearman's correlation coefficients for all pairs of genes in a data set are calculated, then a correlation threshold *T* is selected, and gene pairs with correlation coefficients higher than *T* are connected to construct a coexpression network. In the *K*-nearest neighbor method (34), after pair-wise spearman's correlation coefficient calculation, for each gene, all other genes are ranked based on their correlation coefficients with the gene, and then a coexpression network is constructed by connecting the *K* mutual nearest neighbors. The ARACNE method (5) calculates the mutual information (MI) for all gene pairs and then estimates the significant levels for MIs. After filtering out gene pairs with insignificant MIs, the method examines each gene triplet among the significant gene pairs and removes one edge based on the following criterion:

$$MI\left(G_{\text{i}},\;G_{\text{j}}\right)\leq \min\left[MI\left(G_{\text{i}},\;G_{\text{k}}\right),\;MI\left(G_{\text{k}},\;G_{\text{j}}\right)\right]\times \left(1-\tau \right)$$

If the tolerance τ is 0, the gene pair with the smallest MI will be removed from the gene triplet. If the tolerance τ is 1, all gene pairs in the gene triplet will be kept. Following Margolin *et al.* (35), the tolerance τ value was set between 0 and 0.15 to provide a reasonable tradeoff between sensitivity and specificity.

##### Construction of a Consensus Coexpression Network

To increase robustness against errors in data, a bootstrapping procedure (5) was included in all network construction methods to generate consensus coexpression networks. Specifically, tumor samples were randomly sampled from the original data set with replacement and assembled into a new bootstrapped data set containing the same number of samples as the original data set. For each data set, we repeated this process 100 times and generated 100 bootstrapped data sets. Next, each of the three network construction methods was used to generate 100 bootstrapped coexpression networks based on 100 bootstrapped data sets. The edges from the 100 bootstrapped coexpression networks were then combined to calculate a support score for each gene pair based on the following formula:

$$S\left[i\right]=\{\begin{array}{l} 1\;gene\;pair\;\epsilon \;Network\;i\\ 0\;gene\;pair\;\notin \;Network\;i \end{array}\;Support\;score=\sum_{i=1}^{100}S\left[i\right]$$

Then, the statistical significance of each gene pair was calculated based on the following formulas suggested in (5):

$$z=\frac{support\;score-\text{mu}}{sigma}$$

$$mu=\sum_{i=1}^{100}\frac{\#Edge\left[i\right]}{\#Total\;Edge}$$

$$sigma=\sqrt[{2}]{\sum_{i=1}^{100}\frac{\#Edge\left[i\right]}{\#Total\;Edge}\times \left(1-\frac{\#Edge\left[i\right]}{\#Total\;Edge}\right)}$$

where *#Edge*[*i*] represents the number of edges for the bootstrap network *i* and *#TotalEdge* represents the number of unique edges among all 100 bootstrap networks. *Z* score was then transformed to the *p* value by comparing with the standard normal distribution. Two genes with a *p* value less than 1 × 10^−6^ were then connected by an edge to construct a consensus coexpression network.

##### Quantification of the Functional Relevance of a Coexpression Network

We used likelihood ratio (LR) to quantify the functional relevance of a coexpression network based on the sets of gold standard functionally similar and dissimilar gene pairs described above. Specifically,

$$LR=\frac{P\left(S|N\right)/P\left(D|N\right)}{P\left(S\right)/P\left(D\right)}$$

where *P*(*S*|*N*) and *P*(*D*|*N*) denote the frequencies of functionally similar (*S*) and dissimilar (*D*) gene pairs, respectively, in the coexpression network (*N*), whereas *P*(*S*) and *P*(*D*) denote all functionally similar and dissimilar gene pairs, respectively, in our gold standard sets.

##### Selection of Parameters for the Three Methods

To construct a coexpression network from a gene expression matrix, we need to set parameters *T*, *K,* and τ for the value-based method, *K*-nearest neighbor method and ARACNE method, respectively. We tested different values of the parameters *T* (from 0.40 to 0.80, step by 0.05), *K* (from 0.1%×D to 1%×D, step by 0.1%×D, D is the number of genes in the data set) and τ (0, 5%, 10%, and 15%). A more stringent parameter can usually lead to higher LR of the constructed network (supplemental Fig. S2 and supplemental Table S1). Although higher LR indicates higher functional relevance of the constructed network and is thus preferred, stringent parameters also lead to more isolated nodes and reduced network coverage (supplemental Fig. S3). To balance the tradeoff between functional relevance and coverage, a series of parameters were tested for each method and the most stringent parameters that produced no more than 10% isolated node were selected.

##### Selection of the Threshold for Identifying Functionally Similar and Dissimilar Gene Pairs

In the section “identification of functionally similar and dissimilar gene pairs”, we selected the top and bottom 1%, 5%, 10%, 15%, 20%, and 25% of all ranked gene pairs as the candidate functionally similar and dissimilar gene pairs. Although a more stringent threshold (*e.g.* top and bottom 1%) could lead to higher discriminant power of the gold-standard data sets, the robustness of LR calculation could be compromised when the number of gene pairs in the gold standard data sets is small. To find a stringent threshold that can produce robust result for each of the cancer type, we calculated the LRs of the 100 bootstrapped networks for each of the cancer type and then computed the coefficient of variations (CVs) of the LRs.

##### Comparison of the Three Network Construction Methods

For each of the cancer type, consensus mRNA coexpression networks were generated using the three methods with the selected parameters. Based on the functionally similar and dissimilar gene pairs from GO, we calculated the LRs of the consensus networks and selected the method with the highest LR to construct coexpression networks.

##### Construction of Protein Coexpression Networks

For the proteomics data set from each of the three cancer types, we first generated 100 bootstrapped data sets using the same sets of samples as those in the 100 bootstrapped mRNA data sets (see the section “Construction of a consensus coexpression network”). Coexpression networks for each of the 100 bootstrapped data sets were constructed using the method and the parameter selected based on the mRNA data sets. The protein coexpression consensus network was then constructed based on the method described in the section Construction of a Consensus Coexpression Network.

##### Edge Level Comparison Between mRNA and Protein Coexpression Networks

Based on the functionally similar and dissimilar gene pairs defined above, we calculated the LRs of mRNA and protein coexpression networks. For each coexpression network, we generated 1000 random networks with the same number of nodes and edges and calculated LRs of these random networks as negative controls. For each coexpression network, we also built a protein-protein interaction network for genes in the coexpression network based on curated protein-protein interactions from the iRef database (38) to serve as a positive control or benchmark.

To quantify the edge-level similarity between the mRNA and protein networks, we calculated the Dice coefficient scores (39).

To test the effect of sample size on LRs of the constructed networks, we performed down-sampling experiments. For each of the three cancer types, we randomly selected *n* samples from mRNA or protein data (*n* was from 10 to 70, with a step increment of 5). Then, we generated the consensus coexpression networks based on the selected samples and calculated LRs based on the functionally similar and dissimilar gene pairs. This process was repeated 100 times.

##### Module Level Comparison Between mRNA and Protein Coexpression Networks

We used the NetSAM package (40) (http://bioconductor.org/packages/release/bioc/html/NetSAM.html) to identify hierarchical modules from the mRNA and protein coexpression networks. Then, we performed the following analyses to compare mRNA and protein modules.

##### Comparison of the Functional Coherence Between mRNA and Protein Modules

We first calculated the *p* values of enrichment for all GO biological process terms for each module using the hypergeometric test (41) and then adjusted the *p* values based on the Benjamini and Hochberg method (42). We selected the smallest adjusted *p* value as the measurement of the functional coherence of the module. Finally, we grouped the modules from each of mRNA and protein networks in each cancer type into three groups: a significant group (adjusted *p* value ≤ 0.01), a marginally significant group (0.01 <adjusted *p* value ≤ 0.15), and an insignificant group (adjusted *p* value > 0.15).

##### Evaluation of the Conservation Level of the mRNA Modules in Corresponding Protein Networks

We first counted the overlapping edges between mRNA and protein networks in each cancer type. Then, we calculated the statistical enrichment of overlapping edges in each mRNA module based on the following hypergeometric test:

$$p=1-\sum_{i=0}^{k-1}\frac{\left(\begin{array}{c} m\\ i \end{array}\right)\;\left(\begin{array}{c} M-m\\ N-i \end{array}\right)}{\left(\begin{array}{c} M\\ N \end{array}\right)}$$

where *M*, *N*, *m* and *k* represent the number of edges in the mRNA network, the number of all overlapping edges, the number of edges in a module, and the number of overlapping edges in the module, respectively. Third, the *p* values were adjusted based on the Benjamini and Hochberg method (42). Finally, the conservation level of a mRNA module in corresponding protein network was measured by -log_10_ (adjusted *p* value).

##### Comparison of the Cytogenetic Band Coherence Between mRNA and Protein Modules

The cytogenetic band information was downloaded from BioMart website (http://www.ensembl.org/biomart/, Ensemble Genes 82 and Homo sapiens genes GRCh28.p3). The analyses were performed the same as described for the functional coherent analysis, replacing GO biological process terms with cytogenetic bands.

##### Visualizing the Impact of Chromosome Colocalization on mRNA and Protein Coexpression

We first calculated the spearman's correlation between each pair of mRNAs or proteins. Then, we ordered the mRNAs or proteins based on their chromosome location and visualized the pair-wise correlation scores in a heat map.

##### Gene Function Prediction Based on the mRNA and Protein Coexpression Networks

We compared mRNA and protein coexpression networks for their gene function prediction potential for a wide variety of GO biological processes and KEGG pathways. The KEGG pathway data set was downloaded using REST-style KEGG API (http://rest.kegg.jp/link/hsa/pathway). Network-based gene function prediction was performed using the well-established random walk-based network propagation algorithm (43), and prediction performance was evaluated using 5-fold cross validation and quantified on the basis of the area under the receiver operating characteristic curve (AUROC). Because the random walk algorithm can only be applied to connected networks, our analyses were based on the maximum component for each of the six coexpression networks instead of the full networks. Thus, networks in this section refer to the maximum component of the full networks.

##### Gold-standard Positive and Negative Gene Sets

For a selected network and a selected GO biological process or KEGG pathway term, genes annotated to the term and also included in the network were defined as a positive gene set and other genes in the network constituted the negative gene set for the GO or KEGG term. Only positive gene sets with at least 20 genes and no more than 10% of the total number of genes in the network were included in our study. For each cancer type, only GO and KEGG terms selected by both the mRNA and protein networks were included in the comparative analysis. According, we had 1673, 1236, and 997 GO terms and 125, 84, and 60 KEGG terms as gold-standard for breast, colorectal, and ovarian cancer, respectively (see supplemental Table S2).

##### Random Walk Analysis

The random walk analysis exploits the global structure of a network by simulating the behavior of a random walker on a network. Given a network with *n* nodes and a set of *k* “seed” gene (*k* > 0), we used the random walk with restart (RWR) technique (43) to calculate a priority score for each gene in the network based on the steady state probability of the random walker staying at the gene, which is formally defined as the following equation:

$$p^{t+1}=\left(1-r\right)Wp^{t}+rp^{0}$$

where the initial vector *p*^0^ of size *n* was constructed such that an equal probability of 1/*k* was assigned to the *k* seed genes, while a probability of 0 was given to all other *n–k* genes in the network, *r* is the restart probability (we set *r* as 0.5 in this paper), *W* is the column-normalized adjacency matrix of the network, and *p^t^* is a vector of size *n* where the *i*-th element holds the probability of being at gene *i* at time step *t*.

The final score of a gene in the network was defined by iterating the above equation until Σ_*i*=1_^*n*^|*p*_*i*_^*t*+1^ − *p_i_^t^*| fell below the predefined threshold of 1 × 10^−6^, as previously described (43, 44). A higher score of a gene represents a closer relationship between the gene and the seed genes.

##### AUROC Calculation

Prediction performance for each selected GO and KEGG term was evaluated using 5-fold cross validation. We first randomly assigned genes in the gold standard positive set into five equal sized subgroups. We kept one subgroup as the testing group, and then combined genes from the other four subgroups as seed genes and calculated the priority scores of all non-seed genes in the network based on the RWR analysis. We ranked all non-seed genes from the highest score to the lowest score and calculated the rank ratio of each gene by dividing its rank by the number of all non-seed genes. Based on the rank ratios of genes in the testing group, we used the R package pROC (https://cran.r-project.org/web/packages/pROC/index.html) to calculate an AUROC score. The above analysis was repeated by using each of the other subgroups as the testing group, and then the mean AUROC was calculated across the 5 folds. A mean AUROC of 1 indicates perfect prediction performance of the network for the GO or KEGG term, whereas a mean AUROC of 0.5 suggest that genes annotated to the GO or KEGG term are randomly distributed in the network.

##### Gene2Net Analysis

We developed Gene2Net, a web-based application that allows users to expand one or multiple genes into a small network and to perform GBA analysis based on the three protein coexpression networks generated from this study. The tool can be accessed from the CPTAC portal in http://cptac.gene2net.org. Briefly, network expansion is based on the random walk analysis described above. GO biological process enrichment analysis for the resulted networks is performed based on hypergeometric test. Gene2Net visualizes expanded networks as interactive node-link diagrams and corresponding enriched GO biological processes in interactive directed acyclic graphs. Using clickable Venn diagrams and sortable heat maps, users can also compare results generated from the three cancer types. All results in Gene2Net can also be downloaded for further analyses. A detailed user manual and a video tutorial are available from the Gene2Net website. For the examples presented in this article, we set the network construction method as Network_Expansion and the number of top ranking neighbors as 10.

## RESULTS

### 

#### 

##### Summary of the mRNA and Protein Profiling Data Sets

Table I summarizes the mRNA and protein profiling data sets used in this study. Gene-level, normalized mRNA profiling data for all three cancer types were downloaded from the TCGA Firehose website (http://gdac.broadinstitute.org). Data for breast and colorectal tumors were generated by RNA-Seq whereas those for ovarian tumors were generated by microarray. Microarray data were used for ovarian cancer because RNA-Seq data were not available for most of the samples analyzed by proteomics. Gene-level, normalized proteomic data for the three cancer types were described in three recent publications (22–24). The three proteomic data sets were generated by different institutes using different proteomic platforms (iTRAQ for breast and ovarian cancers and label-free for colorectal cancer) and normalized by different algorithms carefully selected and justified by individual publications. Including three methodologically diverse datasets in this study increases the generalizability of our results. For each cancer type, only overlapping samples and overlapping genes with both mRNA and protein abundance data were included in our analysis. Accordingly, breast, colorectal, and ovarian cancer analyses were based on 77 samples and 5988 genes, 87 samples and 3764 genes, and 174 samples and 2988 genes, respectively.

**Table I:** Summary of the mRNA and protein profiling data sets

Cancer type	mRNA				Protein				Matched data	
	Sample #	Gene #	Technology	Data source	Sample #	Gene #	Technology	Data source	Sample #	Gene #
Breast	1058	20501	Illumina HiSeq 2000	Firehose	77	6281	iTRAQ	Ref 23	77	5988
Colorectal	264	20501	Illumina HiSeq 2000	Firehose	90	3899	label-free	Ref 22	87	3764
Ovarian	541	17814	Agilent 244K	Firehose	174	3327	iTRAQ	Ref 24	174	2988

##### Comparison of Methods for Coexpression Network Construction

Using the mRNA profiling data sets from the three cancer types, we compared three methods that are widely used for mRNA coexpression network construction, including: the value-based method (33), the *K*-nearest neighbor method (34), and the ARACNE method (35). For each method, the most stringent parameter that produced no more than 10% isolated node in the constructed network (supplemental Fig. S3) was used for this study. The bootstrapping procedure (5) was included in all methods to build consensus networks that are more robust to sampling variability and errors in the data.

To evaluate the functional relevance of the networks constructed by different methods, we derived “gold-standard” by calculating pair-wise semantic similarity for all genes based on GO biological process, molecular function, and cellular component annotations, respectively. For each type of GO annotation, the top 5% of gene pairs with the highest similarity scores were designated as a positive set of functionally similar gene pairs whereas the bottom 5% were designated as a negative set of functionally dissimilar gene pairs. The 5% threshold was selected to maximize the discriminant power of the gold-standard sets while ensuring the robustness of the evaluation results (supplemental Fig. S4).

To compare the functional relevance of the networks constructed by the three methods, we computed the LRs of neighboring genes being functionally similar *versus* dissimilar using the gold standard sets. As shown in supplemental Fig. S5, the *K*-nearest neighbor method resulted in the highest LRs for all cancer types and all GO ontologies, except for the combination of ovarian cancer and biological process, where the value-based method had the highest LR. Accordingly, we selected the *K*-nearest neighbor method for coexpression network construction in this study and the optimal *K* was 12, 11, and 9 for breast, colorectal, and ovarian cancer, respectively (supplemental Fig. S3).

##### mRNA and Protein Coexpression Networks are Wired Very Differently

Based on the *K*-nearest neighbor method and the optimal *Ks*, we construct both mRNA and protein coexpression networks for the three cancer types (supplemental Tables S3–S9). For breast cancer (5988 genes), the mRNA coexpression network had 22,940 edges whereas the protein coexpression network had 19,925 edges. These numbers were 10,713 and 13,110 for colorectal cancer (3764 genes), and 7815 and 6091 for ovarian cancer (2988 genes).

**Fig. 1.**
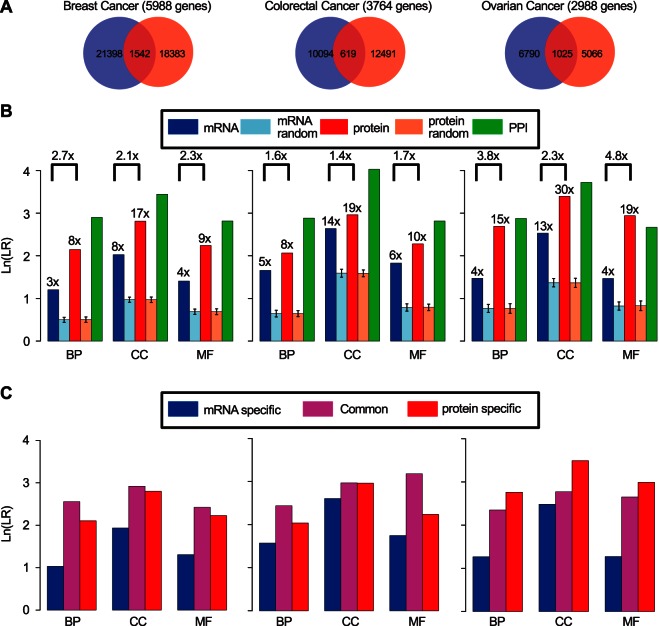
**Edge level comparison between mRNA and protein coexpression networks of the three cancer types.** *A*, Edge overlap between mRNA coexpression network (*blue*) and protein coexpression network (*red*). *B*, The likelihood ratios (*LRs*) calculated for individual networks with gold-standard reference data sets derived from GO biological process (*BP*), cellular component (*CC*) and molecular function (*MF*) annotations, respectively. *Blue*, *light blue*, *red*, *light red* and *green bars* represent mRNA coexpression network, mRNA random network, protein coexpression network, protein random network, and protein-protein interaction (*PPI*) network, respectively. *C*, The LRs of mRNA specific edges (*blue*), protein specific edges (*red*), and common edges (*magenta*).

As shown in Fig. 1*A*, there was very limited overlap in edges between mRNA and protein coexpression networks for all three cancer types (Dice coefficient < 0.15, p≈1 for overlapping significance based on the Fisher's exact test). The overlap remained low (Dice coefficient < 0.25) even when *K* was increased to 599, 376, and 299 for breast, colorectal, and ovarian cancers, respectively (*i.e.* up to 10% of all genes in a network were considered as direct neighbors for each gene, supplemental Fig. S6). These results indicate that mRNA and protein coexpression networks are wired very differently.

##### Protein Networks are More Closely Aligned with Function

Both mRNA (blue bars) and protein (red bars) coexpression networks were more likely to connect functionally similar gene pairs than dissimilar gene pairs, with LRs ranging from 3 to 30 (Fig. 1*B*). These values were significantly higher than those calculated for corresponding random networks with the same numbers of nodes and edges (light blue and light red bars, *p* < 2.2e-16). Deviation of the LRs of the random networks from 1 (log likelihood ratio = 0) can be explained by the enrichment of genes quantified by both technologies in certain GO terms.

Protein networks showed higher (1.4-fold to 4.8-fold) LRs than corresponding mRNA networks (Fig. 1*B*). As depicted in Fig. 1*C*, for breast and colorectal cancers, common edges shared by both mRNA and protein coexpression networks showed the highest LRs (magenta bars), followed closely by edges specific to protein coexpression networks (red bars). For ovarian cancer, edges specific to protein coexpression networks outscored the common edges. For all three cancer types, edges specific to mRNA coexpression networks (blue bars) showed the lowest LRs. Notably, the LRs of the protein coexpression networks approached those of the benchmark networks constructed based on curated protein-protein interactions from the iRef database (38) (green bars, Fig. 1*B*). These data quantitatively demonstrate that protein coexpression networks are highly functionally relevant, and they are more closely aligned with function than mRNA coexpression networks.

**Fig. 2.**
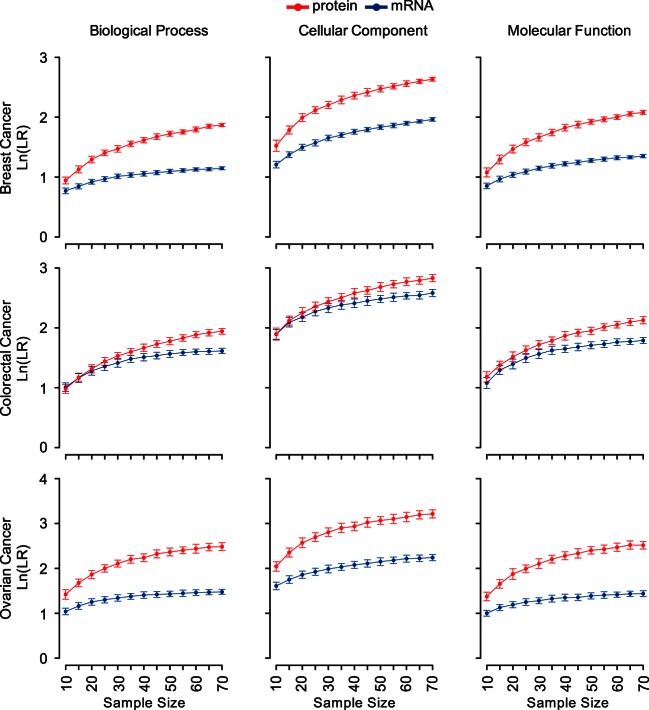
**Sample size effect on the functional relevance of coexpression networks.** *x* axis represents the numbers of samples in down-sampling analyses and *y* axis represents the average values of the natural logarithm transformed LRs for 100 coexpression networks generated by randomly selected samples for different sample sizes. Each *error bar* represents the S.D. of the natural logarithm transformed LRs for one set of 100 coexpression networks. *Red* and *blue lines* represent the protein and mRNA coexpression networks, respectively.

To evaluate the sample size effect on the functional relevance of the constructed mRNA and protein coexpression networks, we further performed down-sampling analysis. As shown in Fig. 2, protein networks had higher LRs than corresponding mRNA networks for all the sample sizes tested, ranging from 10 to 70. Both mRNA and protein coexpression networks had increased LRs with larger sample sizes, but a bigger sample size-dependent LR increase was observed among protein coexpression networks. For all three cancer types, the largest LR increase was found when the sample size went from 10 to 20. Therefore, we recommend a minimal sample size of 20 for protein coexpression network construction.

##### Protein Network Modules are More Functionally Homogeneous

**Fig. 3.**
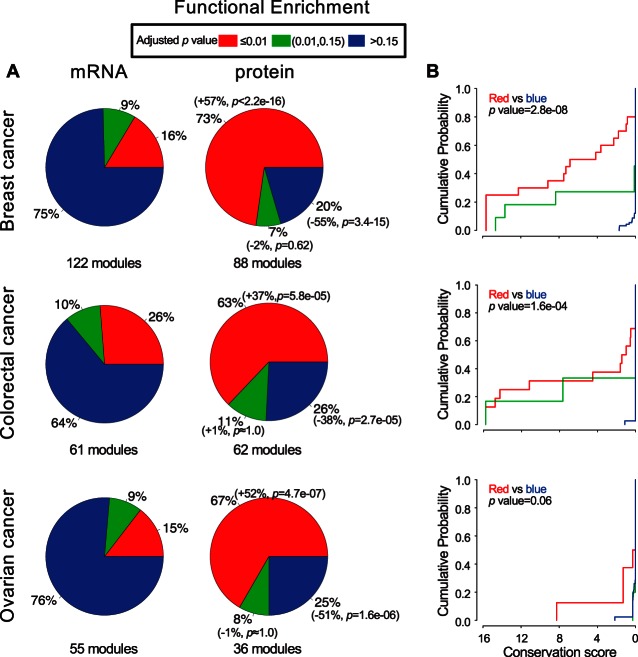
**Functional homogeneity of mRNA and protein coexpression modules.** *A*, Pie charts comparing the functional coherence between mRNA and protein modules from the three cancer types. *Red*, *green* and *blue* represent modules with significant (adjusted *p* value ≤ 0.01), marginally significant (0.01<adjusted *p* value ≤ 0.15), and insignificant (adjusted *p* value > 0.15) GO biological process enrichment, respectively. The total number of modules for each network is provided under each pie chart. The proportion differences between the same colored sections in the mRNA and protein pie charts are indicated in the parentheses beside the proportional number of the protein pie chart. The “+” and “-” signs correspond to higher and lower proportion in the protein pie chart compared with corresponding mRNA pie chart, respectively. The *p* values in the parentheses were calculated by two-sided Fisher's exact test. *B*, Empirical cumulative distribution plots of the conservation scores of mRNA modules in corresponding protein networks for individual module groups. *Line colors* represent the same module groups as in (*A*). The *p* values were calculated by one-sided Kolmogorov-Smirnov test.

We next studied the functional homogeneity of network modules in the coexpression networks. Biological networks usually have a hierarchical modular organization (45) and network modules with tightly connected components are considered as functional blocks of the cell (46). We used the NetSAM algorithm (40) to reveal hierarchical modular architectures of the coexpression networks and then performed enrichment analysis to evaluate the functional coherence of the identified modules (supplemental Tables S10–S15). Among the 122, 61, and 55 mRNA modules identified for breast, colorectal, and ovarian cancer, 16%, 26%, and 15% were enriched in at least one GO biological process (multiple-test adjusted *p* < 0.01, hypergeometric test), respectively. In contrast, among the 88, 62 and 36 protein modules for the three cancer types, significantly larger proportions (73%, 63%, and 67%; *p* < 2.2e-16, *p* = 5.8e-05, and *p* = 4.7e-07, respectively, Fisher's exact test) showed significant GO enrichment (Fig. 3*A*). To examine the conservation level of the mRNA modules in corresponding protein networks, we performed the hypergeometric test for each mRNA module to quantify the statistical enrichment of its edges in the corresponding protein network. As depicted in Fig. 3*B*, functionally coherent mRNA modules (*i.e.* red modules with significant GO enrichment) were more likely to have their edges preserved in corresponding protein networks than functionally incoherent mRNA modules (*i.e.* blue modules with insignificant GO enrichment), with *p* values equal 2.8e-08, 1.6e-04, and 0.06 for breast, colorectal, and ovarian cancer, respectively (one-sided Kolmogorov-Smirnov test).

**Fig. 4.**
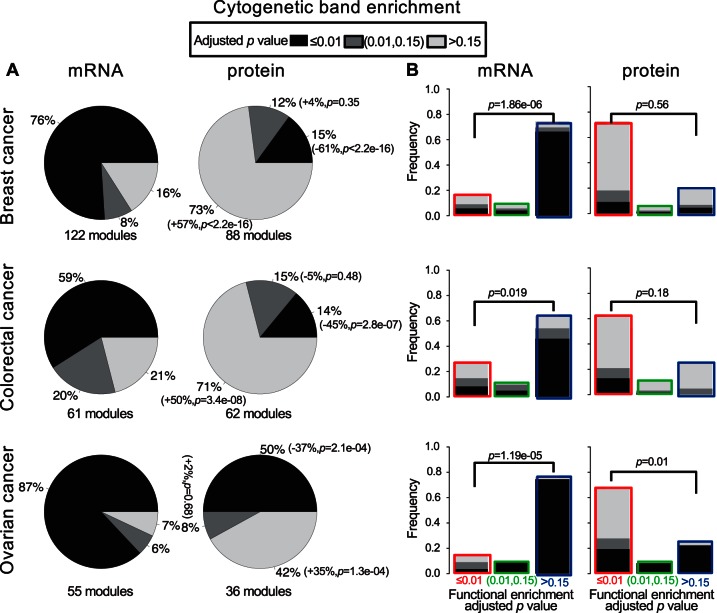
**Impact of chromosome colocalization on mRNA and protein coexpression.** *A*, Pie charts comparing the cytogenetic band enrichment analysis results between mRNA and protein modules from the three cancer types. *Black*, *dark gray* and *light gray colors* represent modules with significant (adjusted *p* value ≤ 0.01), marginally significant (0.01 <adjusted *p* value ≤ 0.15), and insignificant (adjusted *p* value >0.15) cytogenetic band enrichment, respectively. The description of the proportion differences and *p* values in the parentheses can be found in the Fig. 3*A* legend. *B*, Bar charts depict results for individual module groups. The *border colors* of the bars and *x* axis labels represent the same module groups as defined in Fig. 3*A*, with *red*, *green* and *blue border colors* representing modules with significant (adjusted *p* value ≤ 0.01), marginally significant (0.01<adjusted *p* value ≤ 0.15), and insignificant (adjusted *p* value > 0.15) GO biological process enrichment, respectively. *p* values are calculated by the Fisher's exact test.

To further explore other possible biological underpinnings of the coexpression modules, we performed cytogenetic band enrichment analysis for both mRNA and protein modules (Fig. 4*A*). Interestingly, 76%, 59 and 87% of the mRNA modules in breast, colorectal, and ovarian cancers were enriched in at least one cytogenetic band (multiple-test adjusted *p* < 0.01, hypergeometric test), and the ratios were significantly higher in functionally incoherent mRNA modules (89%, 72%, and 98%) than in functionally coherent mRNA modules (35%, 31%, and 25%, *p* < 0.05, Fisher's exact test). For all three cancer types, significantly smaller proportions (15%, 14%, and 50%) of the protein modules showed significant cytogenetic band enrichment compared with the mRNA modules (*p* < 2.2e-16, *p* = 2.8e-07, and *p* = 2.1e-04, respectively, Fisher's exact test). In breast cancer and colorectal cancer, the proportions of the cytogenetic band enriched modules were similar between functional coherent and incoherent protein modules (*p* = 0.56 and 0.18, Fisher's exact test), whereas in ovarian cancer, this proportion was significantly lower in functionally coherent modules than in functionally incoherent modules (29% *versus* 89%, *p* = 0.01, Fisher's exact test). The impact of chromosomal colocalization on mRNA coexpression is obvious for all cancer types when visualizing gene coexpression along human chromosomes, as indicated by the striking red diagonal lines, but such impact was much weaker at the protein level, and the difference was highly statistically significant for most of the chromosomes (supplemental Fig. S7-S9). Taken together, our results demonstrate that protein coexpression modules are primarily driven by functional homogeneity of the genes, whereas chromosomal colocalization plays a significant role in determining mRNA coexpression. Moreover, functionally coherent mRNA modules are preferably preserved in protein networks.

##### Protein Networks Better Predict Biological Functions

**Fig. 5.**
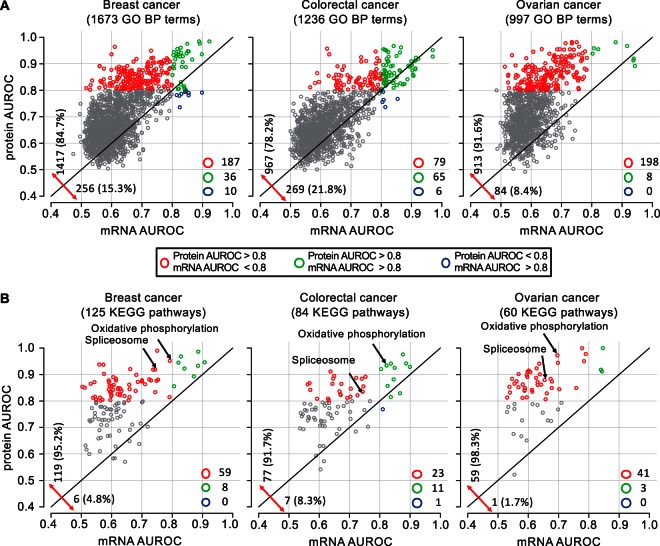
**Gene function prediction based on mRNA and protein coexpression networks of the three cancer types.** *A*, Scatter plots comparing the gene function prediction performance between mRNA and protein coexpression networks of the three cancer types based on GO biological process annotations. GO terms are represented by circles and grouped according to their combination of AUROCs from mRNA and protein networks, as indicated by *different colors. B*, Scatter plots comparing the gene function prediction performance between mRNA and protein coexpression networks of the three cancer types based on KEGG pathway annotations. KEGG pathway terms are represented by circles and grouped according to their combination of AUROCs from mRNA and protein networks, as indicated by *different colors*. Two KEGG pathways previously reported to have poor mRNA-protein correlations are indicated by *arrows*.

To evaluate the relative utility of mRNA and protein profiling data in predicting gene cofunctionality, we compared mRNA and protein coexpression networks for their gene function prediction potential for a wide variety of GO biological processes and KEGG pathways. Network-based gene function prediction was performed using the random walk-based network propagation algorithm (43), and prediction performance was evaluated using 5-fold cross validation and quantified on the basis of the AUROC. GO biological process and KEGG pathway terms were selected for each cancer type separately (supplemental Table S2) based on the size filtering criteria described in Materials and Methods. Protein networks showed better prediction performance compared with corresponding mRNA networks for 85%, 78 and 92% of the GO terms for breast, colorectal, and ovarian cancer, respectively (Fig. 5*A*, supplemental Table S16). Protein networks achieved good performance (AUROC > 0.8) for 223, 144, and 206 GO terms for breast, colorectal, and ovarian cancer, respectively. In contrast, mRNA networks achieved good performance only for 46, 71, and 8 GO terms for the three cancer types, respectively, and the vast majority of these overlapped with the GO terms for which protein networks had good performance (Fig. 5*A*). In the analyses based on KEGG pathway terms, protein networks demonstrated an even more striking advantage compared with mRNA networks, and performance gain was obvious for KEGG pathways previously reported to have poor mRNA-protein correlations (22–24), such as oxidative phosphorylation and spliceosome (Fig. 5*B*, supplemental Table S17). For 23 GO biological process terms and 11 KEGG pathway terms, good performance was achieved by all three protein networks, but by none of the mRNA networks (supplemental Tables S16–S17). These processes and pathways covered a wide range of biological phenomena including cell cycle, tricarboxylic acid cycle, focal adhesion, mRNA surveillance pathway, spliceosome, antigen processing and presentation, gluconeogenesis, regulation of ligase activity, lipid oxidation, mitotic DNA integrity checkpoint, mitochondrial transport, among others. These results show that proteomics data strengthened the connection between gene expression and function for at least 75% of the GO biological processes and 90% of the KEGG pathways. Our analyses also identified specific biological processes and pathways whose assessment would benefit most from direct protein measurements.

##### Protein Networks Reveal Novel Gene-function Relationships

To make the highly functionally relevant breast, colorectal, and ovarian cancer protein coexpression networks available and useful to the broad scientific community, we developed a web application Gene2Net, which allows users to expand one or multiple genes into a small network based on the protein coexpression networks and then perform GBA analysis to generate hypotheses on gene-function relationships. Here we use some examples to illustrate the potential use of the tool.

**Fig. 6.**
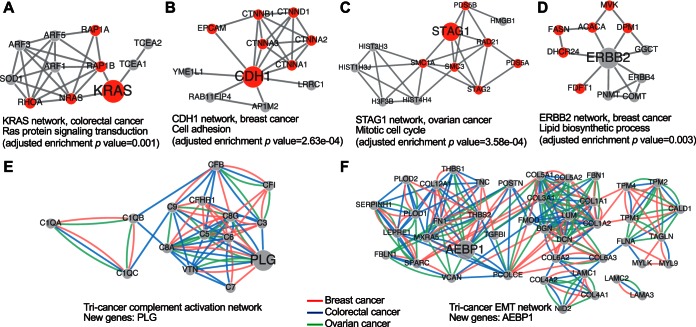
**Protein coexpression network-based inference of gene-function relationship.** *A*, KRAS network in colorectal cancer. The *small nodes* are the top ranking neighbors of KRAS, and *red nodes* represent genes participating in Ras protein signaling transduction. *B*, CDH1 network in breast cancer, and *red nodes* represent genes participating in cell adhesion. *C*, STAG1 network in ovarian cancer, and *red nodes* represent genes participating in mitotic cell cycle. *D*, ERBB2 network in breast cancer. The *small nodes* are the top ranking neighbors of ERBB2, and *red nodes* represent genes participating in lipid biosynthetic process. *E*, Tri-cancer complement activation network. The *small nodes* represent known complement activation genes annotated to the GO term (GO:0006956) and the *large node* represents the common top ranking neighbor across the three cancer types. *Red*, *blue* and *green lines* represent edges from breast cancer, colorectal cancer, and ovarian cancer network, respectively. *F*, Tri-cancer EMT network. The *small nodes* represent known EMT related genes and the *large node* represents the common top ranking neighbor across the three cancer types.

First, we applied the tool to predict functions for all recently published driver genes (47) in the three cancer types (see supplemental Table S18). Among the 126, 39, and 27 drivers that were included in the breast, colorectal, and ovarian cancer networks, we were able to predict functions for 38, 10, and 14 genes, respectively (GO enrichment analysis under FDR 1%, See supplemental Table S19–S22). Previously known GO annotations were identified for 27, 9, and 11 among these genes. For example, using KRAS, a frequently mutated gene in colorectal cancer, to query the colorectal cancer network, we associated the gene to its well-known role in Ras protein signaling transduction (Fig. 6*A*). Using CDH1 to query the breast cancer network, we associated the gene to its well-established role in cell adhesion (Fig. 6*B*). Using STAG1 to query the ovarian cancer network, we associated the gene to known function in cell cycle (Fig. 6*C*). Moreover, new GO annotations were predicted for 21, 6, and 11 of the driver genes in the three cancer types, respectively. Some of these predicted annotations were closely related to existing GO annotations for the genes, but others associated the genes to new biological processes. For examples, using ERBB2 (HER2), a frequently amplified gene in breast cancer, to query the breast cancer network, we associated the gene to lipid biosynthetic process (Fig. 6*D*). Although ERBB2 has not been annotated to this process by GO, ERBB2 positive breast cancers have been shown to produce significantly high amounts of fats and the fat synthetic process is required for survival of ERBB2-positive breast cancer cells (48). Accordingly, knock down of two lipid synthesis genes in our ERBB2 network (*ACACA* and *FASN*) has been shown to significantly decrease cell viability of the ERBB2-positive breast cancer cell line BT474 (48).

In addition to individual genes, gene lists can also be used as input to Gene2Net. To identify new players in complement activation, a central process of cancer immunity (49), we queried each of the three networks using a list of complement activation genes annotated by GO. The three complement activation networks shared 15 common genes (Fig. 6*E*), including a new gene PLG (plasminogen) that was not included in GO annotation. Consistent with this prediction, a recent study showed that PLG can serve as a complement inhibitor in addition to its well-established role in fibrinolysis (50). As another example, we queried each of the three networks using a list of genes curated in MsigDB (6) for the epithelial-mesenchymal transition (EMT), which plays a critical role in promoting metastasis in epithelium-derived carcinomas (51). The three EMT networks shared 42 common genes, including a new gene, AEBP1 (AE binding protein 1, Fig. 6*F*). Interestingly, AEBP1 is one of ten genes in a recently published pan-cancer EMT signature (52).

The above examples demonstrate that our protein coexpression network-based tool allows retrieval of known functions for a gene, prediction of new functions, and identification of new genes for a biological process of interest.

## DISCUSSION

With matched mRNA and protein profiling data from three cancer types, we have performed the first systematic study to investigate the relative utility of mRNA and protein profiling data in predicting gene cofunctionality. Although many studies have reported only a moderate correlation between mRNA and protein profiles (15, 19–22), whether protein profiling data better reflects cellular functions has remained unanswered, because the reported mRNA-protein discrepancy may have both biological and technical explanations (16, 17, 21). Our study provided quantitative evidence to demonstrate that protein profiling data is more closely aligned with function than mRNA profiling data. Proteomic data strengthened the link between gene expression and function for the vast majority of biological processes and pathways. We identified a subset of biological processes and pathways for which protein measurements would be most critical. We also developed Gene2Net, which will allow biologists to generate hypotheses on new gene-function relationships based on the protein coexpression networks.

Although mRNA profiling has been the dominant approach to studying gene expression and its relationship to cellular functions, it has been suggested that genes with similar mRNA expression profiles are not necessarily functionally coupled (11). Our results showed that chromosomal colocalization plays a significant role in determining mRNA coexpression. Somatic copy number alteration may be an important driver of this phenomenon (22–24). In addition, genomic colocalization-driven coexpression has been previously reported in *Caenorhabditis elegans* (53) and *Saccharomyces cerevisiae* (54). Thus, this observation may also be explained by other mechanisms such as colocalization of coexpressed genes in regions of active chromatin or enhancers shared by neighboring genes on chromosomes. The impact of genomic colocalization on gene coexpression is significantly reduced at the protein level than mRNA level. (supplemental Fig. S7–S9). Although mRNA coexpression was driven by both cofunction and chromosomal colocalization of the genes, protein coexpression was driven primarily by functional similarity between coexpressed genes. Importantly, functionally coherent mRNA modules are preferably preserved in protein networks (Fig. 3*B*), suggesting a role of protein level regulation in coordinating gene functions.

Among the three cancer types, proteomic data provided the largest added value for ovarian cancer (Fig. 1*B*, Fig. 3). We note that mRNA profiling data for ovarian cancer were generated by microarray, whereas mRNA data for breast and colorectal cancers were generated by RNA-Seq. The observed differences may be partially attributable to the different platforms. However, this also may reflect the unique biology of ovarian cancer. Prevalent copy number alterations in ovarian cancer (27) may create widespread gene expression alterations at the transcriptome level, thereby requiring more extensive post-transcriptional regulation to buffer against non-functional alterations (11, 55). Indeed, chromosomal colocalization had a much stronger impact on mRNA coexpression in ovarian cancer compared with the other two cancer types, and such impact was reduced, but still visible at the protein level in ovarian cancer (supplemental Fig. S9).

Although current proteomic platforms can identify more than ten thousand proteins, the number of quantifiable proteins remain much smaller than those can be quantified by mRNA profiling. In this study, the quantifiable proteins in the breast, colorectal, and ovarian data sets were 6281, 3899, and 3327, respectively, whereas the number of quantified genes in corresponding mRNA profiling data sets were 20501, 20501, and 17814. Our study was limited to genes with both mRNA and protein abundance measurements. but we believe our conclusion is not biased, because the same trend was observed with the number of studied genes increasing from 2988 in ovarian cancer to 3764 in colorectal cancer and 5988 in breast cancer. Moreover, the robustness of our conclusion was also confirmed by down-sampling experiments using the breast cancer data sets (see supplemental Text S2 and supplemental Fig. S10).

The network topology may affect the priority scores of genes in the network. Zhang *et al.* (44) tried to remove this effect by assessing the statistical significance of the scores. To evaluate whether considering network topology could improve network-based function prediction, we combined two statistic metrics, localP and edgeP, with the rank ratio metric for assessing the significance of the priority scores (see supplemental Text S2). On average, considering network topology only increased the AUROCs less than 2% (supplemental Fig. S11). Furthermore, results based on all three types of AUROCs consistently suggest that protein networks significantly outperformed mRNA networks in gene function prediction.

In conclusion, our results demonstrate that proteome profiling outperforms transcriptome profiling for coexpression based gene function prediction. The GBA strategies developed in transcriptomic studies would be more effective when applied to proteomic data. Gene function and disease studies would benefit immensely from broad adoption of global proteome profiling technologies.

## Supplementary Material

Supplemental Data

## Footnotes

^1^ The abbreviations used are:

GBA: guilt-by-association
TCGA: The Cancer Genome Atlas
CPTAC: the Clinical Proteomic Tumor Analysis Consortium
GO: Gene Ontology
KEGG: Kyoto Encyclopedia of Genes and Genomes
iTRAQ: isobaric peptide labeling approach
LR: likelihood ratio
ARACNE: Algorithm for the Reconstruction of Accurate Cellular Networks.
