# Supplementary material for: Proteome Profiling Outperforms Transcriptome Profiling for Coexpression Based Gene Function Prediction
Source: Mol Cell Proteomics. 2016 Nov 11;16(1):121–34. doi: 10.1074/mcp.M116.060301 (PMC5217778; doi:10.1074/mcp.M116.060301)
Supplement: Supplemental Data [file supp_16_1_121__index.html]

Proteome profiling outperforms transcriptome profiling for co-expression based gene function prediction — Proteome Profiling Outperforms Transcriptome Profiling for Coexpression Based Gene Function Prediction — Comparing Omics Platforms for Gene Function Prediction — Supplemental Data 

# Proteome Profiling Outperforms Transcriptome Profiling for Coexpression Based Gene Function Prediction

## Supplemental Data

- Supplementary figures S1-S11, text S1-S2 and table S1, S3, S18, and S19. (.pdf, 12.8 MB) - Supplementary figures S1-S11, text S1-S2 and table S1, S3, S18, and S19.
- Table S2 (.xlsx, 88 KB) - This spreadsheet lists Gold-standard GO biological process and KEGG pathway terms for individual cancer types.
- Table S4-S9 (.xlsx, 1.3 MB) - This spreadsheet lists mRNA and protein co-expression networks for breast cancer, colorectal cancer and ovarian cancer.
- Table S10-S15 (.xlsx, 79 KB) - This spreadsheet lists functional enrichment results and cytogenetic band results for mRNA and protein co-expression modules of breast cancer, colorectal cancer and ovarian cancer.
- Table S16-S17 (.xlsx, 236 KB) - This spreadsheet lists AUROCs for the gold-standard GO biological process terms and KEGG pathways in the three cancer types.
- Table S20-S22 (.xlsx, 69 KB) - This spreadsheet lists the detailed information of function prediction for the driver genes in the three cancer types based on the Gene2Net.
