# Supplementary material for: Proteome Profiling Outperforms Transcriptome Profiling for Coexpression Based Gene Function Prediction
Source: Mol Cell Proteomics. 2016 Nov 11;16(1):121–34. doi: 10.1074/mcp.M116.060301 (PMC5217778; doi:10.1074/mcp.M116.060301)
Supplement: Supplemental Data [file 10.1074_M116.060301_mcp.M116.060301-1.pdf]

Proteome profiling outperforms transcriptome profiling for co-expression based gene function prediction

Jing Wang, Zihao Ma, Steven A. Carr, Philipp Mertins, Hui Zhang, Zhen Zhang, Daniel W. Chan, Matthew J.C. Ellis, R. Reid Townsend, Richard D. Smith, Jason E. McDermott, Xian Chen, Amanda G. Paulovich, Emily S. Boja, Mehdi Mesri, Christopher R. Kinsinger, Henry Rodriguez, Karin D. Rodland, Daniel C. Liebler, Bing Zhang*

Supplementary materials and methods

Supplementary File 1

This file contains figures S1-S11, text S1-S2 and table S1, S3, S18, and S19.

Supplementary Data Set S1 - Table S2

This spreadsheet lists Gold-standard GO biological process and KEGG pathway terms for individual cancer types.

Supplementary Data Set S2 - Table S4-S9

This spreadsheet lists mRNA and protein co-expression networks for breast cancer, colorectal cancer and ovarian cancer.

Supplementary Data Set S3 - Table S10-S15

This spreadsheet lists functional enrichment results and cytogenetic band results for mRNA and protein co-expression modules of breast cancer, colorectal cancer and ovarian cancer.

Supplementary Data Set S4 - Table S16-S17

This spreadsheet lists AUROCs for the gold-standard GO biological process terms and KEGG pathways in the three cancer types.

Supplementary Data Set S5 - Table S20-S22

This spreadsheet lists the detailed information of function prediction for the driver genes in the three cancer types based on the Gene2Net.

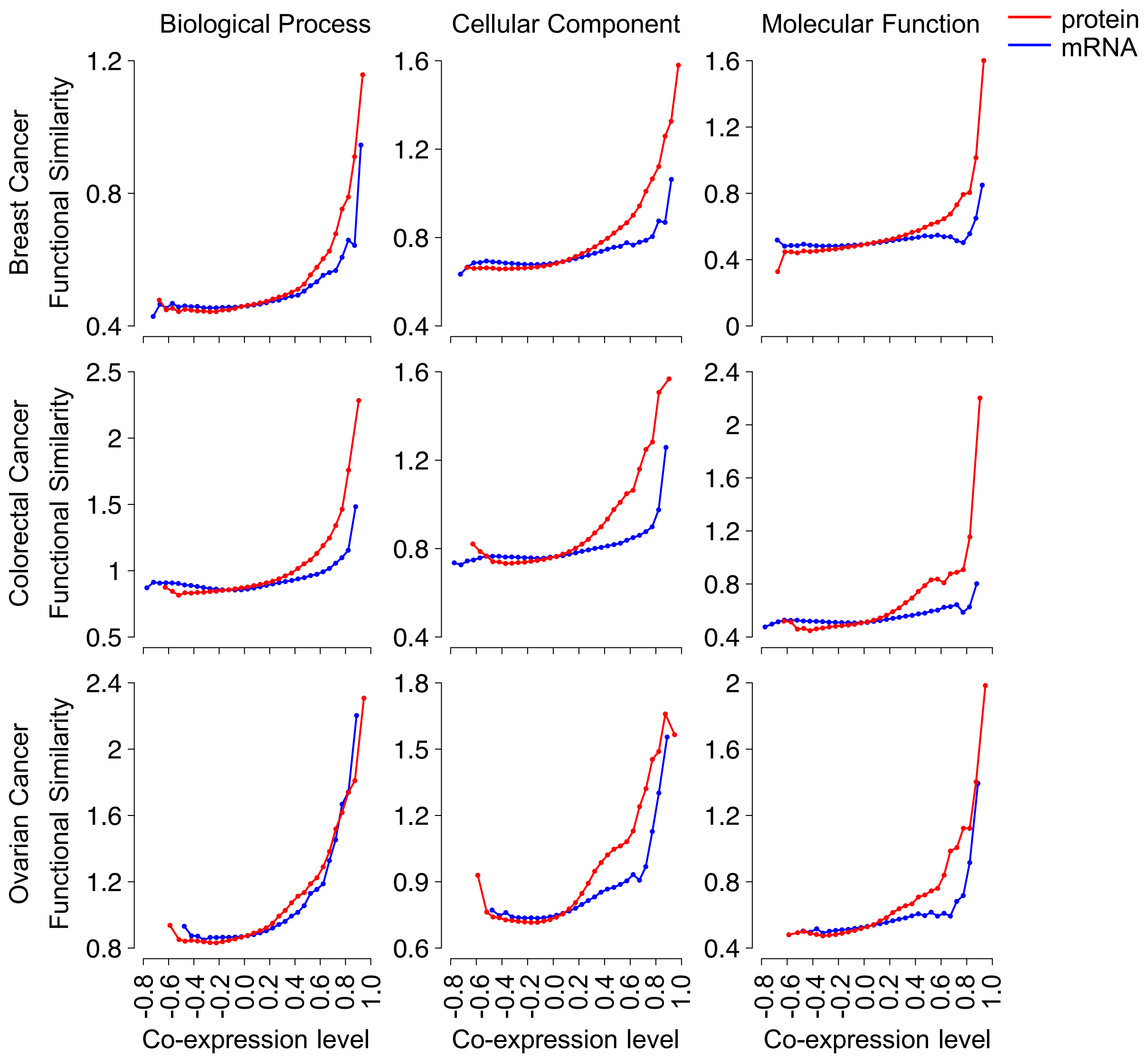

Fig. S1. Relationship between functional similarity and co-expression level of gene pairs. The co-expression level was calculated by the spearman's correlation between mRNA (blue) or protein (red) abundance of each pair of genes from breast cancer, colorectal cancer and ovarian cancer, whereas functional similarity of gene pairs was calculated based on the BMA method (see Method section) according to GO biological process, cellular component and molecular function annotations. The spearman's correlations are binned into 0.05 unit intervals and the average functional similarity for each bin is plotted as a dot.

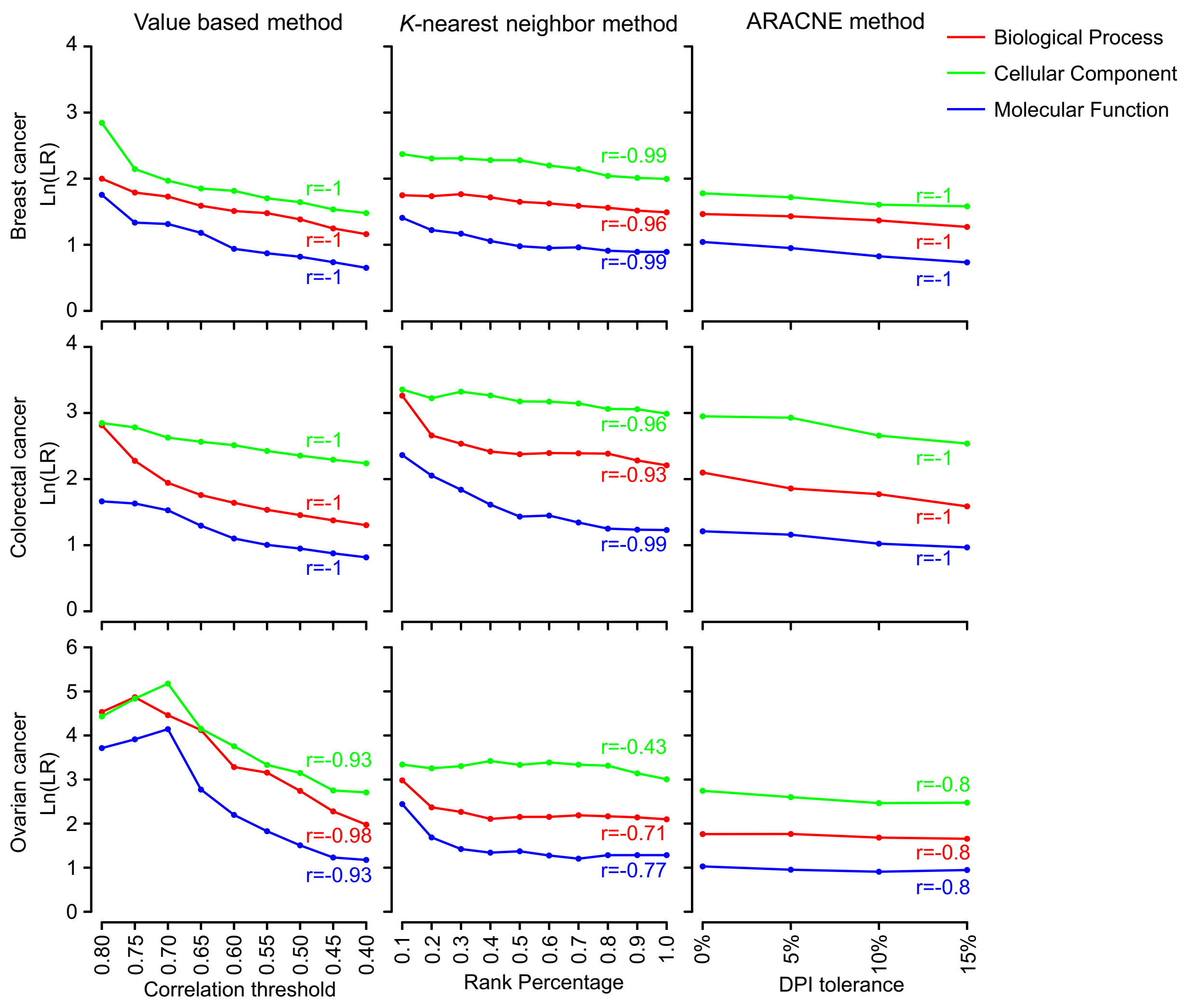

Fig. S2. Relationship between parameter settings and likelihood ratio (LR). Each row represents one of the three cancer types and each column represents one of the three methods. X-axis of each plot represents the values of the tested parameters whereas y-axis of each plot represents the natural logarithm transformed LRs calculated for individual networks based on GO biological process (BP, red), cellular component (CC, green) and molecular function (MF, blue) annotations, respectively. This analysis used the top 1% and bottom 1% gene pairs as gold standard sets of functionally similar and dissimilar gene pairs, respectively (see Materials and Method); the same trend was observed when the threshold was increased to 5%, 10%, 15%, 20% and 25% (Table S1).

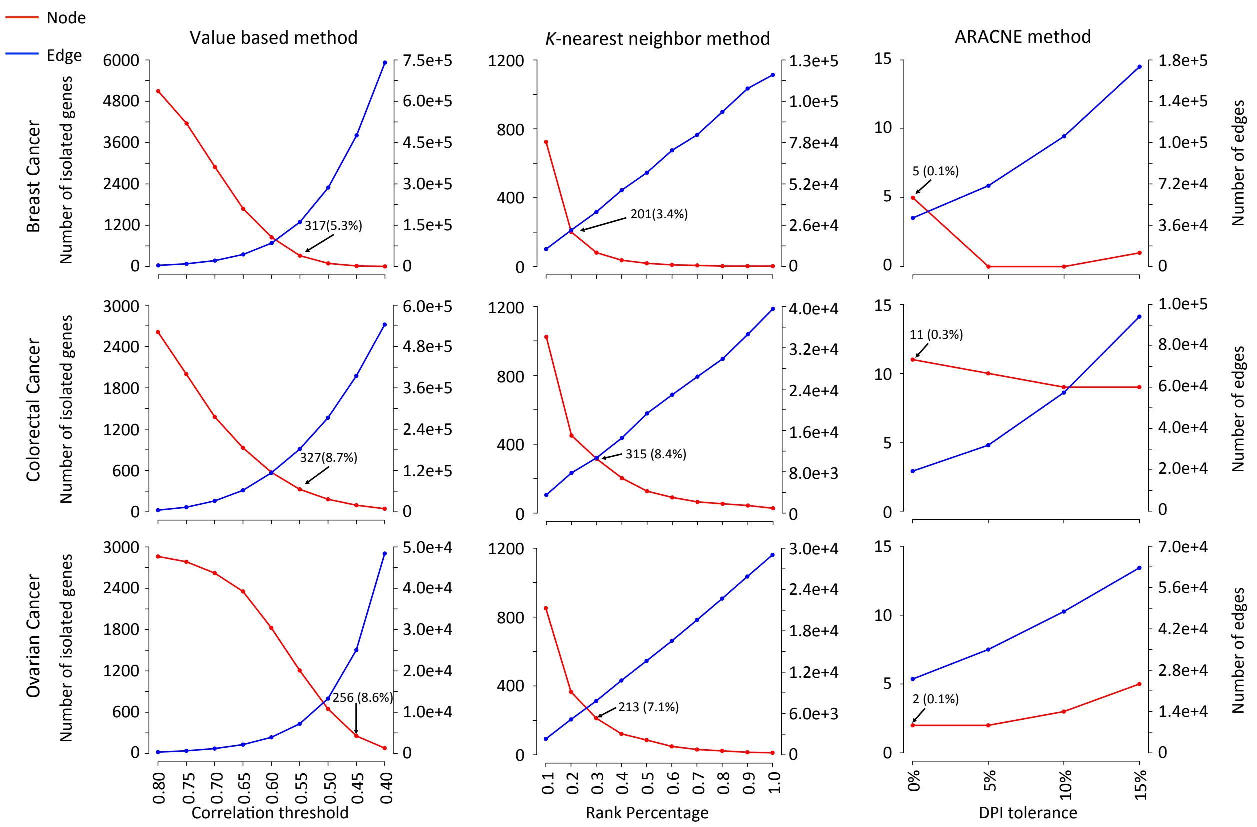

Fig. S3. Parameter selection for the three network construction methods. Each row represents one of the three cancer types and each column represents one of the three methods. X-axis of each plot represents the values of the tested parameters whereas left and right y-axis of each plot represent the numbers of isolated genes and the total numbers of edges, respectively, of the constructed mRNA co-expression networks. The red and blue lines represent the numbers of isolated genes and the total numbers of edges, respectively, for different parameters. The parameters pointed by the black arrows are the ones selected for the study. Specifically, for the value-based method, T was set as 0.55 for breast cancer and colorectal cancer and 0.45 for ovarian cancer. For the K-nearest neighbor method, K was set as 0.2%×D for breast cancer (K=12) and 0.3%×D for colorectal cancer (K=11) and ovarian cancer (K=9). For the ARACNE method, τ was set as 0 for all three cancer types.

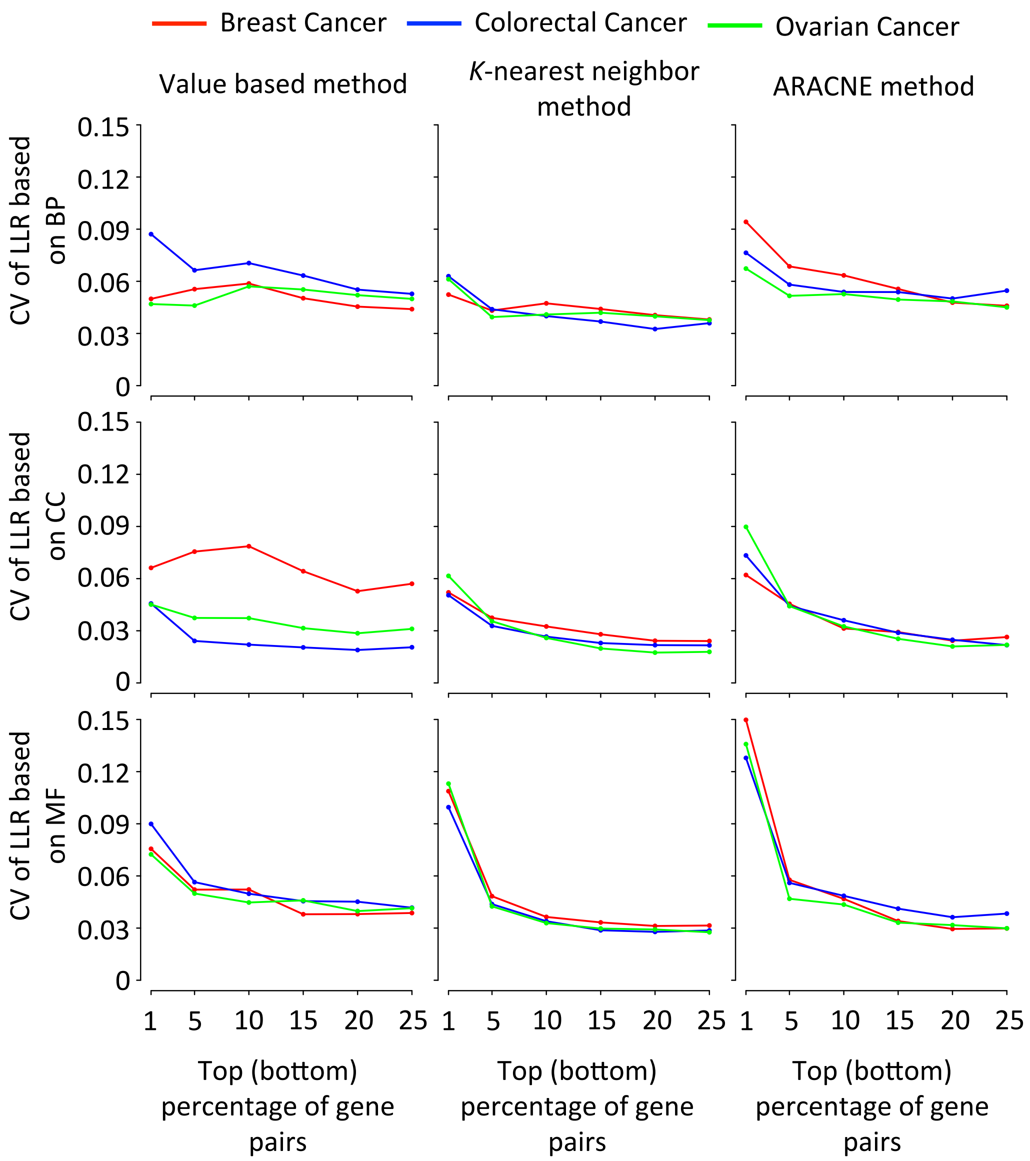

Fig. S4. Threshold selection for identifying functionally similar and dissimilar gene pairs. Each Row represents one of the three GO ontologies (biological process [BP], cellular component [CC], molecular function [MF]) and each column represents one of the three methods. X-axis of each plot represents threshold values and y-axis of each plot represents coefficient of variation (CV) of the likelihood ratios (LRs) of the 100 bootstrapped networks. Red, blue and green lines represent breast cancer, colorectal cancer and ovarian cancer networks, respectively.

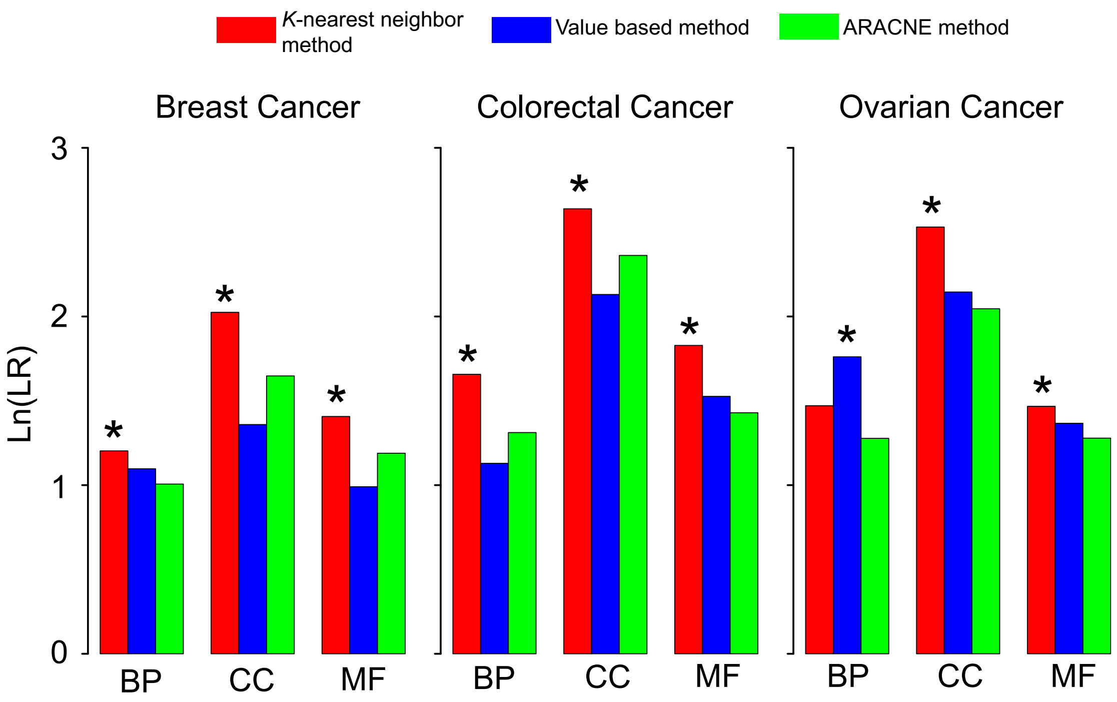

Fig. S5. Comparison of the three network construction methods based on the selected parameters and functionally similar and dissimilar gene pairs. The LRs were calculated for individual networks with gold-standard reference data sets derived from GO biological process (BP), cellular component (CC) and molecular function (MF) annotations, respectively. Red, blue, and green represent the k-nearest neighbor method, the value-based method, and the ARACNE method, respectively. The best method for each cancer and GO combination is highlighted by a "*". Based on the results, the k-nearest neighbor method was selected for the study to compare mRNA and protein networks.

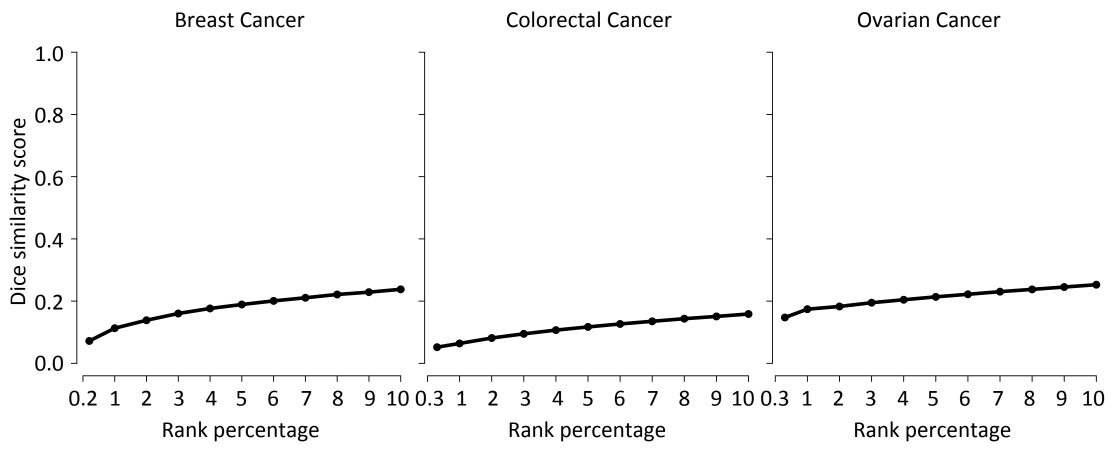

Fig. S6. Impact of the selection of *K* on the Dice coefficient scores quantifying edge similarity between mRNA and protein networks. Each plot compares the result from the selected *K* (12, 11, and 9 for breast, colorectal, and ovarian cancer, respectively, corresponding to the top 0.2%, 0.3%, and 0.3% of all genes in corresponding networks) to those from more relaxed *K*s corresponding to 1% to 10% of all genes in corresponding networks, stepped by 1%.

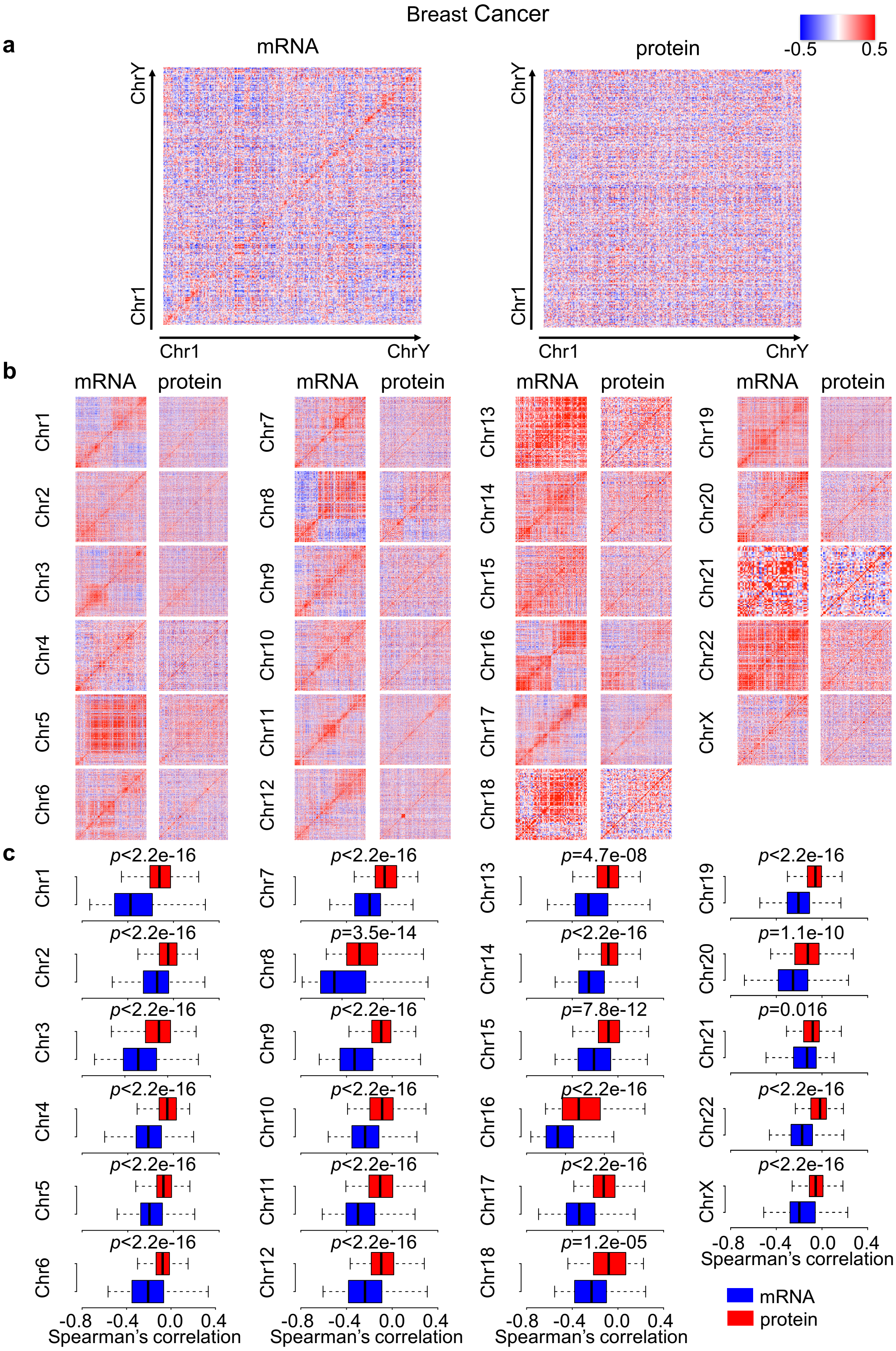

Breast Cancer
a
mRNA
protein
-0.5
0.5
ChrY
Chr1
Chr1
ChrY
b
mRNA
protein
Chr1
Chr2
Chr3
Chr4
Chr5
Chr6
Chr7
Chr8
Chr9
Chr10
Chr11
Chr12
Chr13
Chr14
Chr15
Chr16
Chr17
Chr18
Chr19
Chr20
Chr21
Chr22
ChrX
c
p<2.2e-16
p=3.5e-14
p=4.7e-08
p=7.8e-12
p=1.2e-05
p=1.1e-10
p=0.016
-0.8 -0.4 0.0 0.4
Spearman's correlation
mRNA
protein

Fig. S7. Impact of chromosome co-localization on mRNA and protein co-expression in breast cancer. Each heat map visualizes the pair-wise spearman correlation coefficients between all mRNA pairs or all protein pairs (a) or between all pairs of mRNAs or proteins in each of the 23 chromosomes (b). Because only a few genes on chromosome Y had both mRNA and protein measurements, we did not plot heat maps for chromosome Y. mRNAs and proteins are both ordered based on the chromosomal location of corresponding genes in both the horizontal and vertical directions. Red and blue in the heat map represent positive and negative correlations, respectively, as indicated in the color scale bar. (c) The box plots comparing the distributions of spearman correlations between chromosome distance and mRNA (blue) or protein (red) co-expression correlation of each pair of genes in the same chromosome. *P* values were calculated based on the two-side wilcox rank sum test.

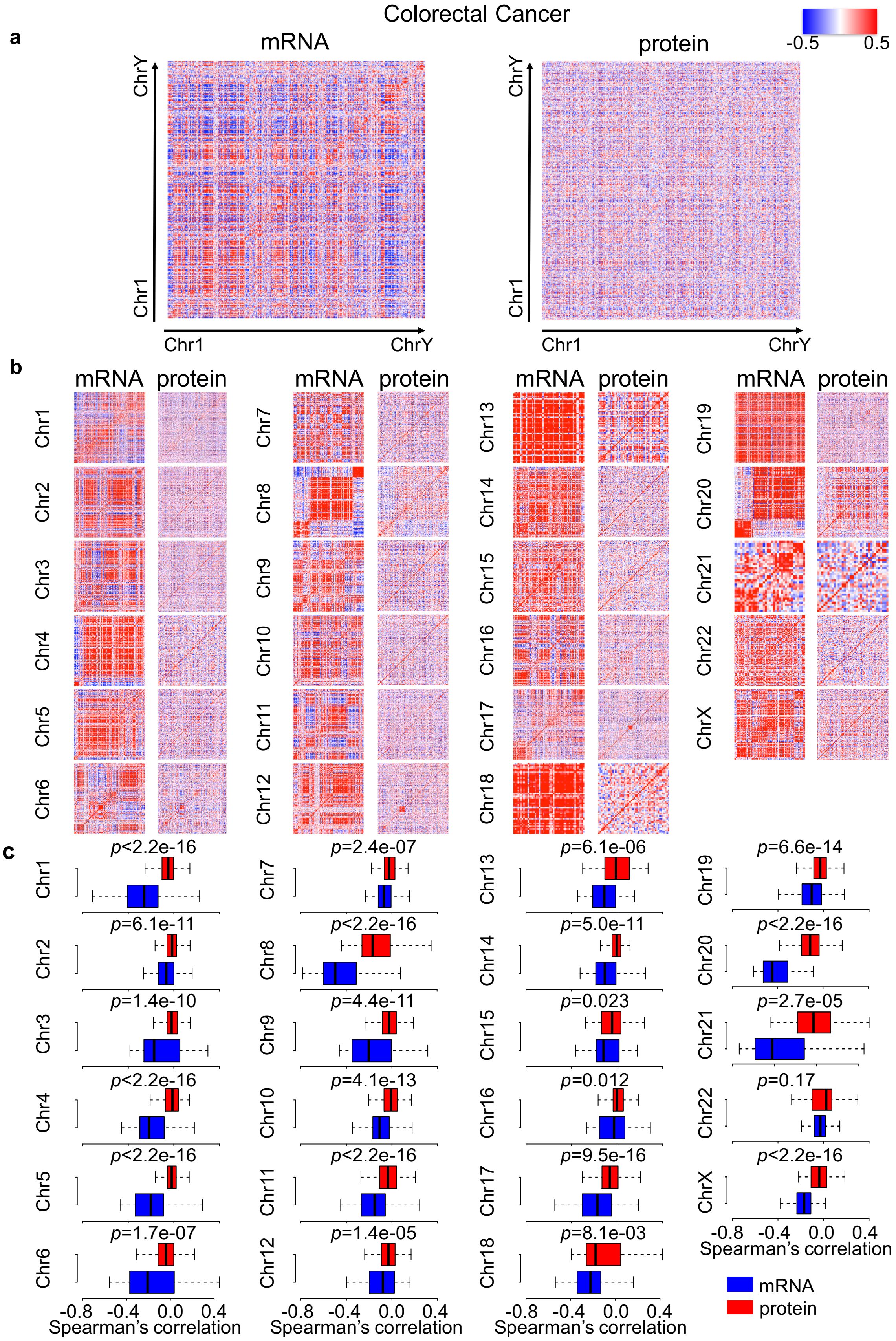
Colorectal Cancer
a
mRNA
protein
-0.5
0.5
ChrY
Chr1
Chr1
ChrY
b
mRNA
protein
Chr1
Chr2
Chr3
Chr4
Chr5
Chr6
Chr7
Chr8
Chr9
Chr10
Chr11
Chr12
Chr13
Chr14
Chr15
Chr16
Chr17
Chr18
Chr19
Chr20
Chr21
Chr22
ChrX
c
p<2.2e-16
p=6.1e-11
p=1.4e-10
p<2.2e-16
p<2.2e-16
p=1.7e-07
p=2.4e-07
p<2.2e-16
p=4.4e-11
p=4.1e-13
p<2.2e-16
p=1.4e-05
p=6.1e-06
p=5.0e-11
p=0.023
p=0.012
p=9.5e-16
p=8.1e-03
p=6.6e-14
p<2.2e-16
p=2.7e-05
p=0.17
p<2.2e-16
-0.8 -0.4 0.0 0.4
Spearman's correlation
mRNA
protein

Fig. S8. Impact of chromosome co-localization on mRNA and protein co-expression in colorectal cancer. Each heat map visualizes the pair-wise spearman correlation coefficients between all mRNA pairs or all protein pairs (a) or between all pairs of mRNAs or proteins in each of the 23 chromosomes (b). Because only a few genes on chromosome Y had both mRNA and protein measurements, we did not plot heat maps for chromosome Y. mRNAs and proteins are both ordered based on the chromosomal location of corresponding genes in both the horizontal and vertical directions. Red and blue in the heat map represent positive and negative correlations, respectively, as indicated in the color scale bar. (c) The box plots comparing the distributions of spearman correlations between chromosome distance and mRNA (blue) or protein (red) co-expression correlation of each pair of genes in the same chromosome. *P* values were calculated based on the two-side wilcox rank sum test.

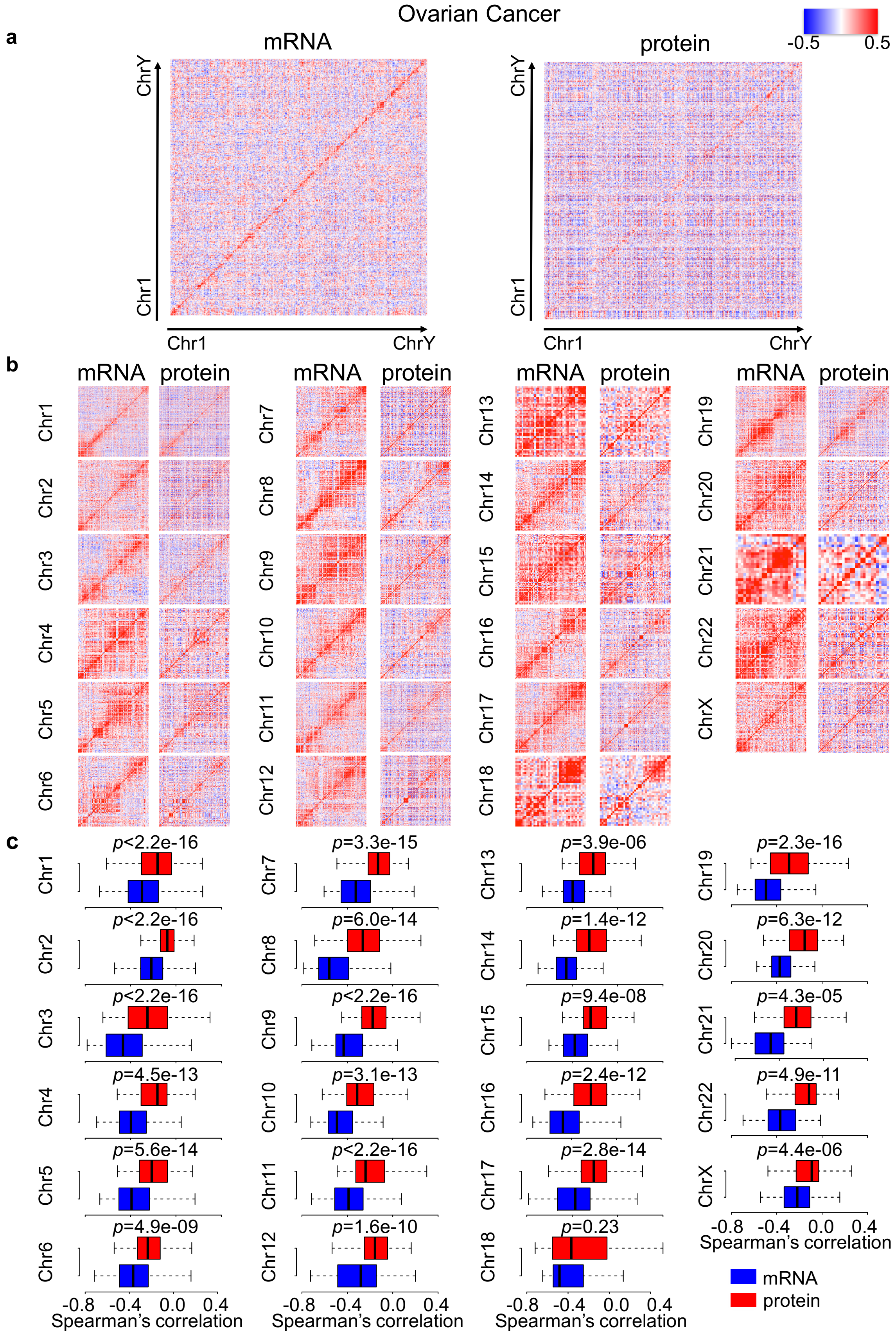
Ovarian Cancer
a
mRNA
protein
-0.5
0.5
ChrY
Chr1
Chr1
ChrY
b
mRNA
protein
Chr1
Chr2
Chr3
Chr4
Chr5
Chr6
Chr7
Chr8
Chr9
Chr10
Chr11
Chr12
Chr13
Chr14
Chr15
Chr16
Chr17
Chr18
Chr19
Chr20
Chr21
Chr22
ChrX
c
p<2.2e-16
p<2.2e-16
p<2.2e-16
p=4.5e-13
p=5.6e-14
p=4.9e-09
p=3.3e-15
p=6.0e-14
p<2.2e-16
p=3.1e-13
p<2.2e-16
p=1.6e-10
p=3.9e-06
p=1.4e-12
p=9.4e-08
p=2.4e-12
p=2.8e-14
p=0.23
p=2.3e-16
p=6.3e-12
p=4.3e-05
p=4.9e-11
p=4.4e-06
-0.8 -0.4 0.0 0.4
Spearman's correlation
mRNA
protein

Fig. S9. Impact of chromosome co-localization on mRNA and protein co-expression in ovarian cancer. Each heat map visualizes the pair-wise spearman correlation coefficients between all mRNA pairs or all protein pairs (a) or between all pairs of mRNAs or proteins in each of the 23 chromosomes (b). Because only a few genes on chromosome Y had both mRNA and protein measurements, we did not plot heat maps for chromosome Y. mRNAs and proteins are both ordered based on the chromosomal location of corresponding genes in both the horizontal and vertical directions. Red and blue in the heat map represent positive and negative correlations, respectively, as indicated in the color scale bar. (c) The box plots comparing the distributions of spearman correlations between chromosome distance and mRNA (blue) or protein (red) co-expression correlation of each pair of genes in the same chromosome. *P* values were calculated based on the two-side wilcox rank sum test.

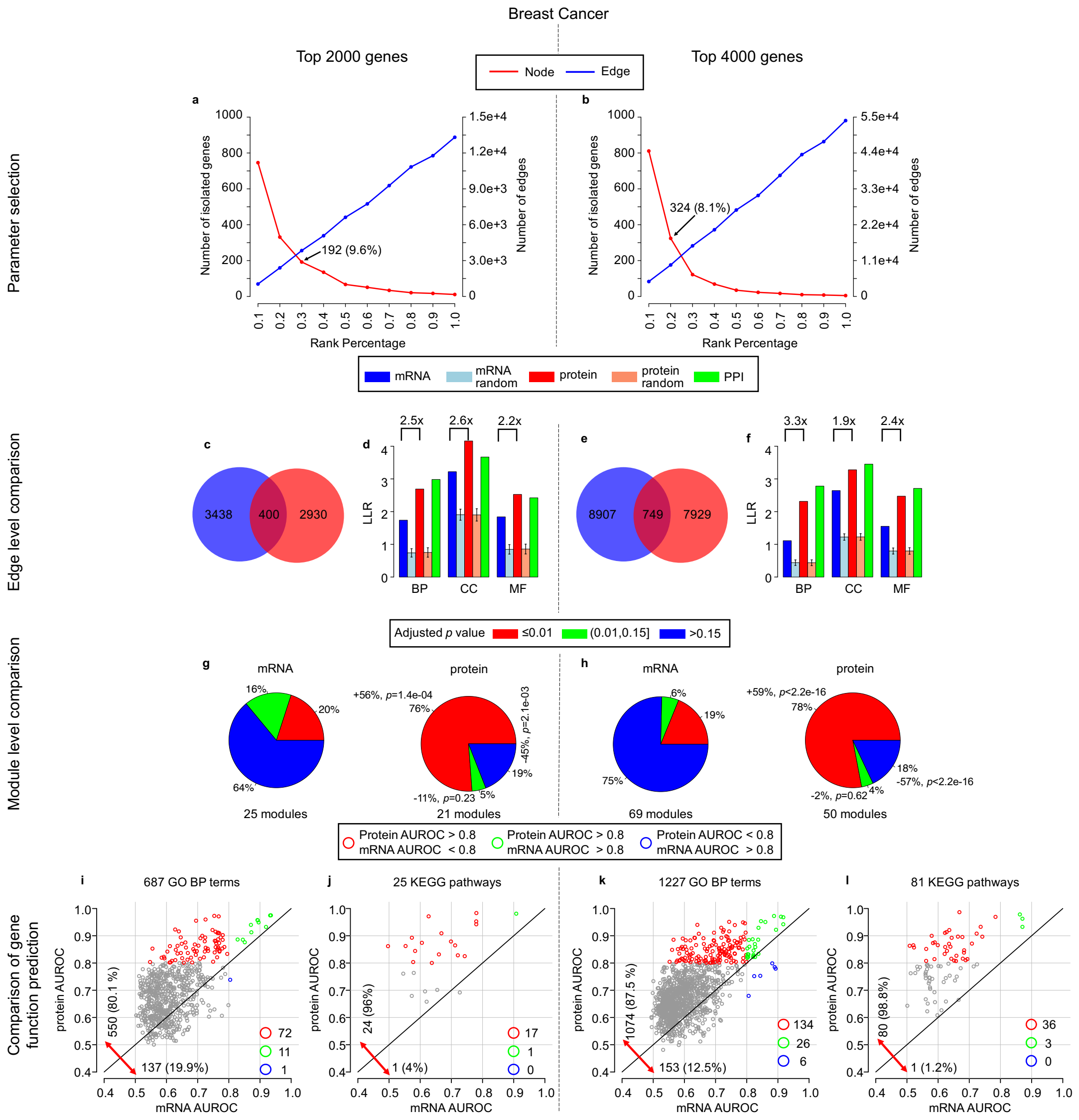

Fig. S10. Downsampling experiments for breast cancer. (a) Optimizing parameter K using RNA-Seq data for the K-nearest neighbor method for the 2000-gene data set. (b-f) Edge level comparison, module level comparison and comparison of gene function prediction between the 2000-gene RNA-Seq and proteomics data sets. (g) Optimizing parameter K using RNA-Seq data for the K-nearest neighbor method for the 4000-gene data set. (h-l) Edge level comparison, module level comparison and comparison of gene function prediction between the 4000-gene RNA-Seq and proteomics data sets. Figure description can be found in the legends of Figs. 1, 3-4 and Fig. S3.

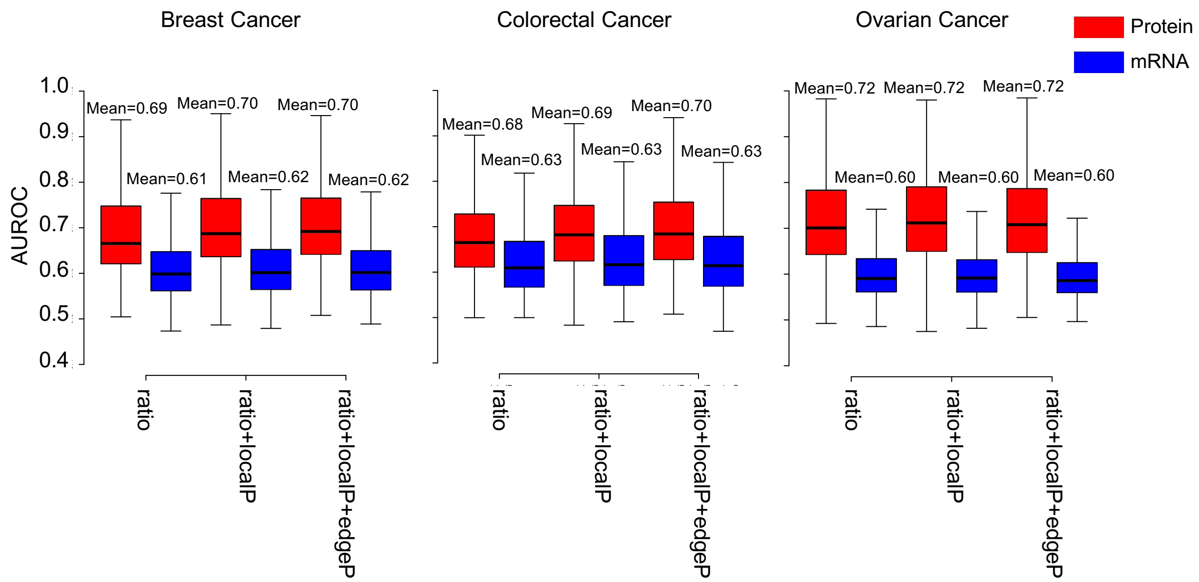

Fig. S11. Comparison of different statistical methods for gene function prediction. To test whether network topology can significantly affect the rank ratio-based AUROCs, we compared the rank ratio-based method to methods that correct for topological bias (ratio+localP and ratio+localP+edgeP) for all tested GO terms. Red and blue represent results from the protein and mRNA co-expression networks, respectively. There was no remarkable difference among the three types of AUROCs at both mRNA and protein levels for all three cancer types, indicating that network topology did not have an obvious impact on the rank ratio-based AUROCs. Furthermore, results based on all three types of AUROCs consistently suggest that protein networks significantly outperformed mRNA networks in gene function prediction.

Text S1

National Cancer Institute Clinical Proteomics Tumor Analysis Consortium (NCI CPTAC)

Steven A. Carr[1], Michael A. Gillette[1], Karl R. Clauser[1], Eric Kuhn[1], D. R. Mani[1], Philipp Mertins[1], Karen A. Ketchum[2], Ratna R. Thangudu[2] , Shuang Cai[2], Mauricio Oberti[2], Amanda G. Paulovich[3], Jeffrey R. Whiteaker[3], Nathan J. Edwards[4], Subha Madhavan[5], Peter B. McGarvey[4], Pei Wang[6], Daniel Chan[7], Akhilesh Pandey[7], Le-Ming Shih[7], Hui Zhang[7], Zhen Zhang[7], Stefani Thomas[7], Heng Zhu[8], Gordon A. Whiteley[9], Steven J. Skates[10], Forest M. White[11], Douglas A. Levine[12], Emily S. Boja1[3], Christopher R. Kinsinger[13], Tara Hiltke[13], Mehdi Mesri[13], Robert C. Rivers[13], Henry Rodriguez[13], Kenna M. Shaw[13], Stephen E. Stein[14], David Fenyo[15], Tao Liu[16], Jason E. McDermott[16], Samuel H. Payne[16], Karin D. Rodland[16], Richard D. Smith[16], Paul Rudnick[17], Michael Snyder[18], Yingming Zhao[19], Xian Chen[20], David F. Ransohoff[20], Andrew N. Hoofnagle[21], Daniel C. Liebler[22], Melinda E. Sanders[22], Zhiao Shi[22], Robbert J. C. Slebos[22], David L. Tabb[22], Bing Zhang[22], Lisa J. Zimmerman[22], Yue Wang[23], Sherri R. Davies[24], Li Ding[24], R. Reid Townsend[24], Matthew J. Ellis[25]

[1]Broad Institute of MIT and Harvard, Cambridge MA 02142

[2]Enterprise Science and Computing, Inc., Rockville, MD 20850

[3]Clinical Research Division, Fred Hutchinson Cancer Research Center, Seattle, WA 98109

[4]Department of Biochemistry and Molecular & Cellular Biology, Georgetown University Medical Center, Washington, DC 20057

[5]Center for Biomedical Informatics, Georgetown University Medical Center, Washington, DC 20057

[6]Icahn School of Medicine at Mount Sinai, New York, NY 10029

[7]Department of Pathology, The Johns Hopkins University, Baltimore, MD 21287

[8]Department of Pharmacology and Molecular Science, The Johns Hopkins University, Baltimore, MD 21287

[9]Antibody Characterization Laboratory, Advanced Technology Program, Inc., Leidos, Frederick, MD 21701

[10]Biostatistics Center, Massachusetts General Hospital Cancer Center, Boston, MA 02114

[11]Department of Biological Engineering, Massachusetts Institute of Technology, Cambridge, MA 02139

[12]Gynecology Service/Department of Surgery, Memorial Sloan-Kettering Cancer Center, New York, NY 10065

[13]National Cancer Institute, Bethesda, MD 20892

[14]National Institute of Standards and Technology, Gaithersburg, MD 20899

[15]Department of Biochemistry, New York University Langone Medical Center, New York, NY 10016

[16]Biological Sciences Division, Pacific Northwest National Laboratory, Richland Washington 99352

[17]Spectragen-Informatics, Rockville, MD 20850

[18]Department of Genetics, Stanford University, Stanford, CA 94305

[19]The Ben May Department for Cancer Research, University of Chicago, Chicago, IL, 60637

[20]University of North Carolina at Chapel Hill, Chapel Hill, NC 27599

[21]Department of Lab Medicine, University of Washington, Seattle, WA 98195

[22]Vanderbilt University School of Medicine, Nashville, TN, 37232

[23]Bradley Department of Electrical and Computer Engineering, Virginia Tech, Arlington, VA

22203

[24]Deparment of Medicine, Washington University in St. Louis, St. Louis, MO 063110

[25]Lester and Sue Smith Breast Center, Dan L. Duncan Comprehensive Cancer Center and Departments of Medicine and Molecular and Cellular Biology, Baylor College of Medicine, Houston, TX, 77030

Text S2

Downsampling experiment

To test whether our conclusion is biased by protein abundance, we performed downsampling experiments using the breast cancer data sets. We estimated protein abundance using the protein length-normalized total precursor intensity (*i.e.* the sum of all precursor MS1-XICs values of all peptides that belong to a protein). The 5988 overlapping genes in the proteomics and RNA-Seq data sets described in the Material and methods were sorted based on the estimated protein abundance. The top 2000 and top 4000 genes with the highest protein abundance were identified to construct two sets of downsampled RNA-Seq and proteomics data, respectively. Following the procedure described in the Material and methods, we identified the optimal parameter *K* in the *K*-nearest neighbor method as 6 and 8 for the 2000-gene and 4000-gene data sets, respectively. Finally, we performed the edge level comparison, module level comparison and comparison of gene function prediction between the downsampled RNA-Seq and proteomics sub-data sets.

Identification of the relationship between network topology and priority scores

To identify whether the network topology affected the priority scores of genes in the network, we assessed the statistical significance of the scores based on the two statistic methods, localP and edgeP. For localP, we kept the same network and let the random walker start from the same number of randomly selected seeds to calculate random scores for each gene. This process was repeated 1000 times to generate a set of random scores. Then, for each gene, a *p* value was estimated by comparing the real score to random scores from the same gene. For edgeP, we kept the same starting genes and used the edge switching method(1) to generate 1000 random networks with the same number of nodes and an identical degree sequence. After generating

1000 random scores for each gene based on the RWR analysis, the *p* value for a gene was estimated by comparing the real score to its random scores. Finally, each gene had three scores: rank ratio, local *p* value and edge *p* value. Then, we defined the minimum of rank ratio and local *p* value as "ratio+localP" and the minimum among three scores as "ratio+localP+edgeP". Using the "ratio+localP" (or "ratio+localP+edgeP") scores and testing group as the input to the R package pROC, we calculated AUROC for each GO term. Thus, each GO term had three AUROC based on rank ratio, "ratio+localP" and "ratio+localP+edgeP". We will compare the difference among these three AUROC for each GO term.

Reference

1. Maslov, S., and Sneppen, K. (2002) Specificity and stability in topology of protein networks. *Science* 296, 910-913

Table S1. Spearman's correlation between candidate parameters of each of the three methods and the resulted likelihood ratios (LRs).

Threshold for candidate gold standard of functionally similar and dissimilar gene pairs	cancer type	method	GO ontology	spearman correlation
Top 1%	Breast	Value	BP	-1
Top 1%	Breast	Value	CC	-1
Top 1%	Breast	Value	MF	-1
Top 1%	Breast	K-nearest neighbor	BP	-0.963636364
Top 1%	Breast	K-nearest neighbor	CC	-0.987878788
Top 1%	Breast	K-nearest neighbor	MF	-0.987878788
Top 1%	Breast	ARACNE	BP	-1
Top 1%	Breast	ARACNE	CC	-1
Top 1%	Breast	ARACNE	MF	-1
Top 1%	Colon	Value	BP	-1
Top 1%	Colon	Value	CC	-1
Top 1%	Colon	Value	MF	-1
Top 1%	Colon	K-nearest neighbor	BP	-0.927272727
Top 1%	Colon	K-nearest neighbor	CC	-0.963636364
Top 1%	Colon	K-nearest neighbor	MF	-0.987878788
Top 1%	Colon	ARACNE	BP	-1
Top 1%	Colon	ARACNE	CC	-1
Top 1%	Colon	ARACNE	MF	-1
Top 1%	OV	Value	BP	-0.983333333
Top 1%	OV	Value	CC	-0.933333333
Top 1%	OV	Value	MF	-0.933333333
Top 1%	OV	K-nearest neighbor	BP	-0.709090909
Top 1%	OV	K-nearest neighbor	CC	-0.43030303
Top 1%	OV	K-nearest neighbor	MF	-0.76969697
Top 1%	OV	ARACNE	BP	-0.8
Top 1%	OV	ARACNE	CC	-0.8
Top 1%	OV	ARACNE	MF	-0.8
Top 5%	Breast	Value	BP	-1
Top 5%	Breast	Value	CC	-0.783333333
Top 5%	Breast	Value	MF	-0.85
Top 5%	Breast	K-nearest neighbor	BP	-0.963636364
Top 5%	Breast	K-nearest neighbor	CC	-1
Top 5%	Breast	K-nearest neighbor	MF	-1
Top 5%	Breast	ARACNE	BP	-1
Top 5%	Breast	ARACNE	CC	-1
Top 5%	Breast	ARACNE	MF	-1
Top 5%	Colon	Value	BP	-1
Top 5%	Colon	Value	CC	-0.233333333
Top 5%	Colon	Value	MF	-0.783333333
Top 5%	Colon	K-nearest neighbor	BP	-0.975757576
Top 5%	Colon	K-nearest neighbor	CC	-0.987878788
Top 5%	Colon	K-nearest neighbor	MF	-0.987878788
Top 5%	Colon	ARACNE	BP	-1
Top 5%	Colon	ARACNE	CC	-1
Top 5%	Colon	ARACNE	MF	-0.8
Top 5%	OV	Value	BP	-1
Top 5%	OV	Value	CC	-0.983333333
Top 5%	OV	Value	MF	-1
Top 5%	OV	K-nearest neighbor	BP	-0.296969697
Top 5%	OV	K-nearest neighbor	CC	-0.854545455
Top 5%	OV	K-nearest neighbor	MF	-0.854545455
Top 5%	OV	ARACNE	BP	-1

Top 5%	OV	ARACNE	CC	-0.6
Top 5%	OV	ARACNE	MF	-0.4
Top 10%	Breast	Value	BP	-1
Top 10%	Breast	Value	CC	0.2
Top 10%	Breast	Value	MF	0.766666667
Top 10%	Breast	K-nearest neighbor	BP	-0.903030303
Top 10%	Breast	K-nearest neighbor	CC	-1
Top 10%	Breast	K-nearest neighbor	MF	-1
Top 10%	Breast	ARACNE	BP	-1
Top 10%	Breast	ARACNE	CC	-1
Top 10%	Breast	ARACNE	MF	-1
Top 10%	Colon	Value	BP	-1
Top 10%	Colon	Value	CC	0.583333333
Top 10%	Colon	Value	MF	-0.633333333
Top 10%	Colon	K-nearest neighbor	BP	-0.951515152
Top 10%	Colon	K-nearest neighbor	CC	-1
Top 10%	Colon	K-nearest neighbor	MF	-1
Top 10%	Colon	ARACNE	BP	-1
Top 10%	Colon	ARACNE	CC	-1
Top 10%	Colon	ARACNE	MF	-0.8
Top 10%	OV	Value	BP	-1
Top 10%	OV	Value	CC	-0.933333333
Top 10%	OV	Value	MF	-0.983333333
Top 10%	OV	K-nearest neighbor	BP	-0.915151515
Top 10%	OV	K-nearest neighbor	CC	-0.927272727
Top 10%	OV	K-nearest neighbor	MF	-0.927272727
Top 10%	OV	ARACNE	BP	-0.4
Top 10%	OV	ARACNE	CC	-0.6
Top 10%	OV	ARACNE	MF	-1
Top 15%	Breast	Value	BP	-1
Top 15%	Breast	Value	CC	0.333333333
Top 15%	Breast	Value	MF	0.183333333
Top 15%	Breast	K-nearest neighbor	BP	-0.939393939
Top 15%	Breast	K-nearest neighbor	CC	-1
Top 15%	Breast	K-nearest neighbor	MF	-1
Top 15%	Breast	ARACNE	BP	-1
Top 15%	Breast	ARACNE	CC	-1
Top 15%	Breast	ARACNE	MF	-1
Top 15%	Colon	Value	BP	-1
Top 15%	Colon	Value	CC	0.683333333
Top 15%	Colon	Value	MF	-0.316666667
Top 15%	Colon	K-nearest neighbor	BP	-0.987878788
Top 15%	Colon	K-nearest neighbor	CC	-0.987878788
Top 15%	Colon	K-nearest neighbor	MF	-0.987878788
Top 15%	Colon	ARACNE	BP	-1
Top 15%	Colon	ARACNE	CC	-1
Top 15%	Colon	ARACNE	MF	-1
Top 15%	OV	Value	BP	-1
Top 15%	OV	Value	CC	-0.883333333
Top 15%	OV	Value	MF	-0.85
Top 15%	OV	K-nearest neighbor	BP	-1
Top 15%	OV	K-nearest neighbor	CC	-0.963636364
Top 15%	OV	K-nearest neighbor	MF	-0.612121212
Top 15%	OV	ARACNE	BP	-0.2
Top 15%	OV	ARACNE	CC	-0.6
Top 15%	OV	ARACNE	MF	-0.8
Top 20%	Breast	Value	BP	-1
Top 20%	Breast	Value	CC	0.333333333
Top 20%	Breast	Value	MF	0.366666667
Top 20%	Breast	K-nearest neighbor	BP	-0.975757576
Top 20%	Breast	K-nearest neighbor	CC	-1
Top 20%	Breast	K-nearest neighbor	MF	-1
Top 20%	Breast	ARACNE	BP	-1
Top 20%	Breast	ARACNE	CC	-1

Top 20%	Breast	ARACNE	MF	-1
Top 20%	Colon	Value	BP	-1
Top 20%	Colon	Value	CC	0.783333333
Top 20%	Colon	Value	MF	-0.1
Top 20%	Colon	K-nearest neighbor	BP	-0.987878788
Top 20%	Colon	K-nearest neighbor	CC	-0.975757576
Top 20%	Colon	K-nearest neighbor	MF	-1
Top 20%	Colon	ARACNE	BP	-1
Top 20%	Colon	ARACNE	CC	-1
Top 20%	Colon	ARACNE	MF	-0.8
Top 20%	OV	Value	BP	-1
Top 20%	OV	Value	CC	-0.816666667
Top 20%	OV	Value	MF	-0.85
Top 20%	OV	K-nearest neighbor	BP	-0.975757576
Top 20%	OV	K-nearest neighbor	CC	-0.951515152
Top 20%	OV	K-nearest neighbor	MF	-0.175757576
Top 20%	OV	ARACNE	BP	-0.8
Top 20%	OV	ARACNE	CC	-0.8
Top 20%	OV	ARACNE	MF	-0.4
Top 25%	Breast	Value	BP	-1
Top 25%	Breast	Value	CC	0.816666667
Top 25%	Breast	Value	MF	0.85
Top 25%	Breast	K-nearest neighbor	BP	-0.975757576
Top 25%	Breast	K-nearest neighbor	CC	-1
Top 25%	Breast	K-nearest neighbor	MF	-1
Top 25%	Breast	ARACNE	BP	-1
Top 25%	Breast	ARACNE	CC	-1
Top 25%	Breast	ARACNE	MF	-1
Top 25%	Colon	Value	BP	-1
Top 25%	Colon	Value	CC	0.9
Top 25%	Colon	Value	MF	0
Top 25%	Colon	K-nearest neighbor	BP	-0.987878788
Top 25%	Colon	K-nearest neighbor	CC	-1
Top 25%	Colon	K-nearest neighbor	MF	-0.987878788
Top 25%	Colon	ARACNE	BP	-1
Top 25%	Colon	ARACNE	CC	-1
Top 25%	Colon	ARACNE	MF	-0.8
Top 25%	OV	Value	BP	-1
Top 25%	OV	Value	CC	-0.683333333
Top 25%	OV	Value	MF	-0.85
Top 25%	OV	K-nearest neighbor	BP	-0.975757576
Top 25%	OV	K-nearest neighbor	CC	-0.963636364
Top 25%	OV	K-nearest neighbor	MF	0.006060606
Top 25%	OV	ARACNE	BP	-0.4
Top 25%	OV	ARACNE	CC	-0.8
Top 25%	OV	ARACNE	MF	-0.4

Table S3. Information of the mRNA and protein co-expression networks (and maximum components) for three cancer types.

Cancer Type	Network type	All Network		Maximum Component	
		#Node	#Edge	#Node	#Edge
Breast Cancer	mRNA	5787	22940	5750	22919
	protein	5791	19925	5756	19907
Colorectal Cancer	mRNA	3449	10713	3363	10652
	protein	3675	13110	3660	13101
Ovarian Cancer	mRNA	2775	7815	2680	7657
	protein	2655	6091	2431	5681

Table S18. Driver genes in the three cancer types

**Breast cancer**
ACO1,ACSL6,ACTB,ACVR1B,AFF4,AHNAK,AKAP9,AKT1,ANK3,APC,AQR,ARFGEF2,ARHGAP35,ARID1A,ARID2,ARID4B,ARNTL,ASH1L,ASPM,ATF1,ATIC,ATM,ATR,BAP1,BCOR,BMPR2,BNC2,BPTF,BRAF,BRCA1,BRCA2,CAD,CARM1,CASP8,CAST,CBFB,CCAR1,CCT5,CDH1,CDK12,CDKN1B,CEP290,CHD4,CHD9,CHEK2,CIC,CLASP2,CLSPN,CLTC,CNOT3,CSDE1,CSNK1G3,CTCF,CUL1,DDX3X,DDX5,DHX15,DIS3,EGFR,EIF1AX,EIF2C3,EIF4A2,EIF4G1,ELF1,EP300,ERBB2,ERBB2IP,ERCC2,FBXW7,FLT3,FMR1,FN1,FOXA1,FOXP1,FUBP1,FUS,G3BP2,GATA3,GOLGA5,GPS2,HCFC1,HLA-A,HLF,HNRPDL,HSPA8,IDH1,ITSN1,KALRN,KDM5C,KEAP1,KLF4,KRAS,LCP1,LPHN2,LRP6,MACF1,MAP2K4,MAP3K1,MAX,MECOM,MED12,MED23,MED24,MGA,MKL1,MLH1,MLL,MLL2,MLL3,MLLT4,MSR1,MTOR,MUC20,MYB,MYH11,MYH14,MYH9,NCOR1,NDRG1,NF1,NF2,NOTCH1,NOTCH2,NR4A2,NRAS,NSD1,NUP107,NUP98,PAX5,PBRM1,PCDH18,PCSK6,PHF6,PIK3CA,PIK3CB,PIK3R1,PIK3R3,PIP5K1A,POLR2B,PRKAR1A,PRKCZ,PTEN,PTGS1,PTPRU,RB1,RBBP7,RBM5,RFC4,RHEB,RPGR,RPL5,RUNX1,SEC24D,SETD2,SETDB1,SF3B1,SFPQ,SMAD4,SMARCA4,SOS1,SOS2,SPTAN1,SRGAP1,STAG1,STAG2,STIP1,STK11,STK4,SUZ12,SVEP1,TAF1,TBL1XR1,TBX3,TCF12,TCF7L2,TFDP1,TGFBR2,THRAP3,TNPO1,TOM1,TP53,TRIO,ZFP36L1,ZFP36L2
**Colorectal cancer**
ACO1,ACSL6,ACVR1B,AKAP9,APC,ARID1A,ARNTL,ASPM,ATM,ATRX,AXIN2,BCOR,BMPR2,BPTF,BRAF,BRWD1,CAD,CASP8,CDC73,CDK12,CDKN1B,CEP290,CHD4,CHD9,CLSPN,CNOT1,CREBBP,CTCF,CTNNB1,CUL1,DIS3,DNMT3A,EGFR,ELF3,FAM123B,FBXW7,FN1,FOXP1,FXR1,GATA3,GNAS,GOLGA5,IDH2,ITSN1,KRAS,LPHN2,MAP2K1,MAP3K4,MECOM,MED12,MED24,MGA,MLL2,MSR1,MYH10,NF1,NR2F2,NR4A2,NRAS,NTN4,NUP107,NUP98,PCBP1,PIK3CA,PIK3R1,POLR2B,PPP2R1A,PTEN,PTGS1,PTPN11,PTPRU,RAD21,RBM10,RTN4,RUNX1,SF3B1,SMAD2,SMAD4,SMC1A,SOS2,SOX9,SRGAP3,STAG2,SYNCRIP,TAF1,TBX3,TCF12,TCF7L2,TGFBR2,TP53,TP53BP1,TRIO,WIPF1,WT1,ZC3H11A
**Ovarian cancer**
ACO1,ACTG1,AFF4,ARID1A,ASH1L,ASPM,ATF1,ATIC,ATR,ATRX,BAP1,BAZ2B,BMPR2,BRAF,BRCA1,BRCA2,CASP1,CCAR1,CCT5,CDK12,CHD1L,CHD4,CLASP2,CLSPN,CSDE1,CTNNB1,CUL2,DDX5,DLG1,DNMT3A,EIF2AK3,EIF4A2,ERBB2IP,F8,FAM123B,FBXW7,FLT3,FMR1,GNAS,GOLGA5,GPS2,HDAC3,HGF,HSP90AA1,ITSN1,KRAS,LPHN2,MAP3K4,MAP4K3,MECOM,MED12,MKL1,MLH1,MLL2,MYH10,NCKAP1,NDRG1,NF1,NOTCH1,NR4A2,NRAS,NSD1,PIK3CA,POLR2B,PTEN,RB1,RHOA,SETD2,SETDB1,SIN3A,SOS1,STAG1,STAG2,TBX3,TCF7L2,TFDP1,TGFBR2,TJP1,TOM1,TP53,TP53BP1,TRIO,YBX1

Table S19. Summary of function prediction for driver genes in the three cancer types

	#Drivers	#Drivers in the network	#Drivers with enriched functions	#Drivers with enriched known functions	#Drivers with enriched new functions
**Breast cancer**	184	126	38	27	21
**Colorectal cancer**	95	39	10	9	6
**Ovarian cancer**	83	27	14	11	11
